# NRP1 interacts with endoglin and VEGFR2 to modulate VEGF signaling and endothelial cell sprouting

**DOI:** 10.1038/s42003-024-05798-2

**Published:** 2024-01-19

**Authors:** Swati Sharma, Marcelo Ehrlich, Manqi Zhang, Gerard C. Blobe, Yoav I. Henis

**Affiliations:** 1 Department of Neurobiology, George S. Wise Faculty of Life Sciences, Tel Aviv University, Tel Aviv, 6997801 Israel; 2 Shmunis School of Biomedicine and Cancer Research, George S. Wise Faculty of Life Sciences, Tel Aviv University, Tel Aviv, 6997801 Israel; 3 Department of Medicine, Duke University Medical Center, Durham, NC 27708 USA; 4 Department of Pharmacology and Cancer Biology, Duke University Medical Center, Durham, NC 27708 USA

**Keywords:** Growth factor signalling, Tumour angiogenesis

## Abstract

Endothelial cells express neuropilin 1 (NRP1), endoglin (ENG) and vascular endothelial growth factor receptor 2 (VEGFR2), which regulate VEGF-A-mediated vascular development and angiogenesis. However, the link between complex formation among these receptors with VEGF-A-induced signaling and biology is yet unclear. Here, we quantify surface receptor interactions by IgG-mediated immobilization of one receptor, and fluorescence recovery after photobleaching (FRAP) measurements of the mobility of another coexpressed receptor. We observe stable ENG/NRP1, ENG/VEGFR2, and NRP1/VEGFR2 complexes, which are enhanced by VEGF-A. ENG augments NRP1/VEGFR2 interactions, suggesting formation of tripartite complexes bridged by ENG. Effects on signaling are measured in murine embryonic endothelial cells expressing (MEEC^+/+^) or lacking (MEEC^-/-^) ENG, along with NRP1 and/or ENG overexpression or knockdown. We find that optimal VEGF-A-mediated phosphorylation of VEGFR2 and Erk1/2 requires ENG and NRP1. ENG or NRP1 increase VEGF-A-induced sprouting, becoming optimal in cells expressing all three receptors, and both processes are inhibited by a MEK1/2 inhibitor. We propose a model where the maximal potency of VEGF-A involves a tripartite complex where ENG bridges VEGFR2 and NRP1, providing an attractive therapeutic target for modulation of VEGF-A signaling and biological responses.

## Abstract (web summary)

A mechanistic study provides insight into complex formation between neuropilin-1, endoglin and VEGFR2 as well as the functional consequences of such associations in VEGF-A induced angiogenesis.

## Introduction

Sprouting angiogenesis is a process where new blood vessels are formed from preexisting vasculature. It plays critical roles in embryogenesis, wound healing and multiple diseases, including cancer^1,2^. Activated endothelial cells (ECs) proliferate and migrate toward the angiogenic stimulus, assemble into solid cords, and subsequently acquire a lumen^3^. Among the vascular endothelial growth factors (VEGFs), the VEGF-A_165_ isoform (hereafter VEGF-A) is most prominent in inducing ECs sprouting followed by angiogenesis^4^. These effects are mediated *via* the VEGF receptors VEGFR1 and VEGFR2, of which VEGFR2 (KDR, kinase insert domain receptor) appears to be the major regulator of most of the VEGF-A-induced signaling pathways^5–7^.

VEGF-A binds to several receptors and co-receptors, including VEGFR2 and neuropilin-1 (NRP1)^8,9^. It binds to the Ig-like domains 2 and 3 of VEGFR2 *via* residues encoded by VEGF-A exons two to five^9–11^, enhancing VEGFR2 dimerization, thus enabling cross-phosphorylation and activation of multiple cell signaling cascades^12–14^. VEGF-A binding to NRP1 occurs *via* the exon 8-encoded C-terminal region of the cytokine^15,16^. In ECs, phosphorylated VEGFR2 (pVEGFR2) initiates multiple downstream signaling pathways, including extracellular signal-regulated protein kinases (Erk1/2), phospholipase C-γ (PLC-γ), phosphoinositide 3-kinase (PI3K)/protein kinase B (Akt), p38 mitogen-activated protein kinases (p38), focal adhesion kinase (FAK), and mammalian target of rapamycin (MTOR)^14,17,18^. NRP1, which lacks catalytic activity of its own, is a transmembrane (TM) protein with a short cytoplasmic tail^19^, which undergoes homo-dimerization^20,21^. It acts as a co-receptor for VEGF-A, which bind to NRP1 and VEGFR2 simultaneously^22–24^. NRP1 was shown to modulate VEGF-A-mediated signaling to migration, survival and three-dimensional sprouting of ECs^25–27^.

Endoglin (ENG), also known as CD105, is a transforming growth factor-β (TGF-β) co-receptor which is expressed in ECs and some additional cell types^28^, and was shown to form homodimers^29,30^. Mutations in ENG lead to hereditary hemorrhagic telangiectasia (HHT), an autosomal dominant vascular disease^31,32^. ENG dysfunction contributes to tumor associated angiogenesis and inflammation^33–36^. Moreover, tumor-associated angiogenesis is induced by angiogenic factors, including VEGF and ENG, which are upregulated under hypoxic conditions^37–40^. These reports indicate that ENG contributes to VEGF-induced angiogenesis, suggesting that interactions between ENG, VEGFR2 and/or NRP1 may provide a mechanism to regulate angiogenesis. Indeed, ENG was shown to interact with NRP1^41^, and its binding to VEGFR2 was found to contribute to VEGF-A-mediated angiogenesis in ECs^42^. However, most studies on the interactions between these receptors were limited to semi-quantitative co-immunoprecipitation, and the nature and dynamics of the complexes between the full-length ENG, NRP1 and VEGFR2 situated in the plasma membrane of live cells were not characterized, leaving the mechanism of how they cooperate to induce VEGF-A signaling and biological outcome unclear.

In the current study, we explored the interactions between ENG, NRP1 and VEGFR2, their modulation by ligand (VEGF-A), and the effects on signaling and biological response (EC vascular sprouting). To measure quantitatively complex formation and dynamics between the above receptors at the surface of living cells, we employed patch/FRAP (fluorescence recovery after photobleaching of one receptor and its modulation by crosslinking and immobilization of another coexpressed receptor), which we have utilized earlier to study the interactions between multiple full-length TGF-β superfamily receptors^30,43–47^. Our studies demonstrated the formation of stable complexes between ENG/NRP1, ENG/VEGFR2, and NRP1/VEGFR2. The interactions between all these receptor pairs were enhanced by VEGF-A. Of note, ENG enhanced the interactions between NRP1 and VEGFR2, which did not compete with each other for binding to ENG. This suggests formation of tripartite complexes where ENG bridges between NRP1 and VEGFR2. Studies on VEGF-A signaling to pVEGFR2 and pErk1/2 in murine embryonic endothelial cells (MEECs) from wild-type (WT) mice expressing ENG (MEEC^+/+^) and from ENG-null mice (MEEC^-/-^)^48^, along with overexpression or siRNA knockdown of *NRP1*, demonstrated that optimal stimulation of pVEGFR2 and pErk1/2 by VEGF-A requires coexpression of ENG and NRP1 with VEGFR2. The ability of VEGF-A to induce sprouting of MEECs paralleled the effects on signaling^3,48^. We propose a model where a tripartite complex comprised of VEGFR2, NRP1 and ENG enforces VEGF-A-mediated signaling, modulating its effects on sprouting of ECs. This has potential implications for the future development of therapies aimed at modulation of VEGF-A signaling and biological responses.

## Results

### ENG, NRP1 and VEGFR2 form stable complexes with each other, which are enhanced by VEGF-A

Sprouting and angiogenesis of ECs involves VEGFR2, NRP1 and ENG. Interactions between pairs of these proteins have been reported^21,41,42^. However, most studies employed co-immunoprecipitation, which is semi-quantitative and detects only complexes that withstand the immunoprecipitation conditions, and did not investigate the formation of triple complexes between these receptors and their potential effects on signaling and EC biology. Therefore, here we endeavored to measure quantitatively the formation and dynamics of complexes between all these receptors by patch/FRAP^43,47^, their modulation by VEGF-A, and the corresponding effects on VEGF-A signaling and biological responses.

**Fig. 1:**
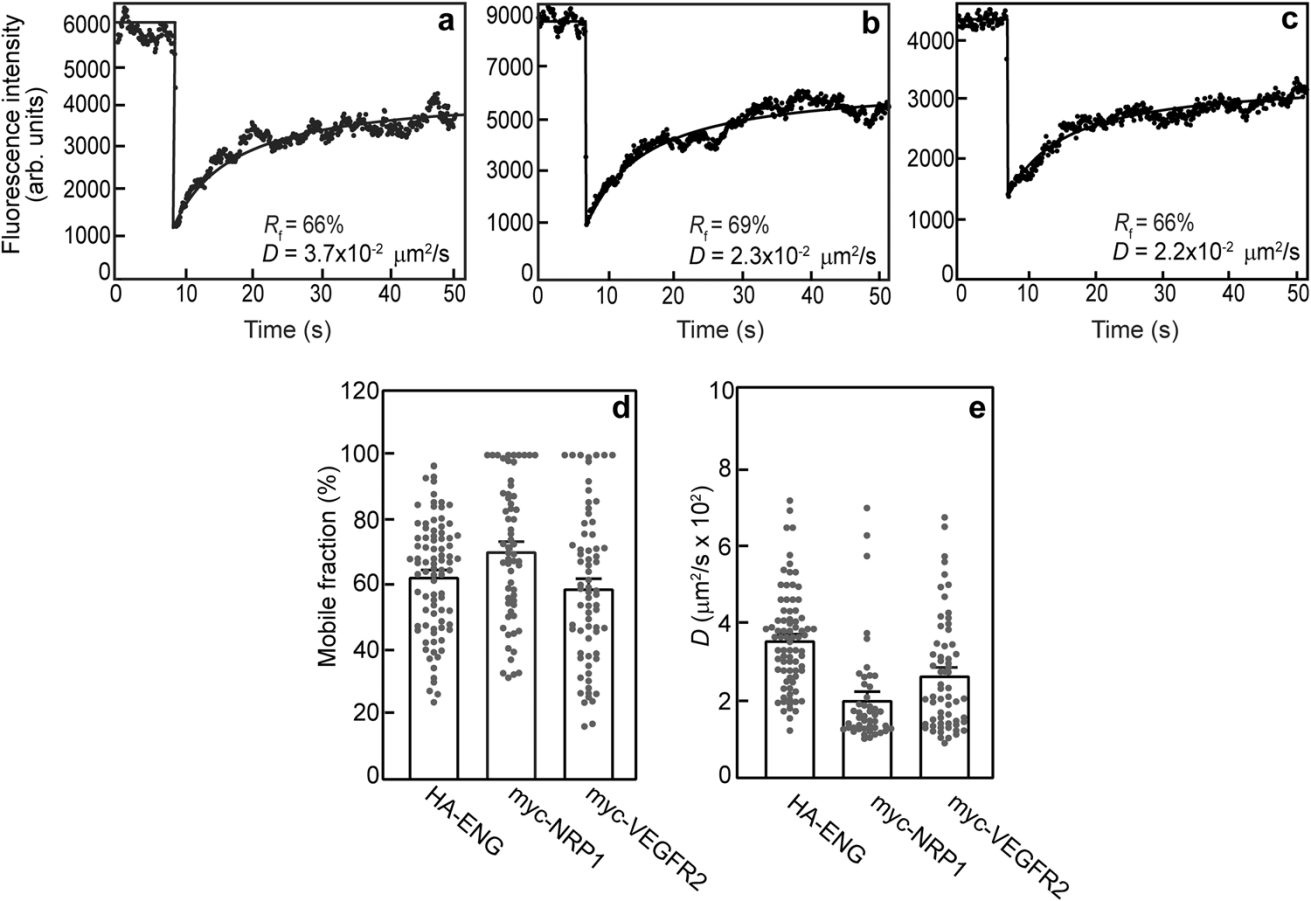
Characterization of the lateral diffusion of ENG, NRP1 and VEGFR2. COS7 cells were transfected by vectors encoding HA-ENG, myc-NRP1 or myc-VEGFR2. At 24 h post-transfection, the cell-surface receptors on live cells were labeled at 4 °C by monovalent fluorescent Fab’ fragments (see Methods), and subjected to FRAP studies conducted at 15 °C to minimize internalization (Methods). Representative FRAP curves showing the lateral diffusion of singly-expressed HA-ENG (**a**), myc-NRP1 (**b**), or myc-VEGFR2 (**c**). Solid lines represent the best-fit (by non-linear regression) to the lateral diffusion equation (see Methods). The *R*_f_ and *D* values for each representative experiment are shown within each panel. Average *R*_f_ (**d**) and *D* values (**e**) derived from multiple patch/FRAP measurements. Bars depict the mean ± SEM of multiple experiments conducted each on a different cell. Some of these numbers are lower in panel e because FRAP curves yielding less than 20% recovery can be accurately analyzed only for *R*_f_.

We first conducted FRAP studies to measure the lateral diffusion of the receptors investigated in the current study. To this end, we expressed ENG, NRP1 or VEGFR2 carrying an extracellular epitope tag (myc or HA) in COS7 cells (the system used to characterize the lateral diffusion of ENG and its interactions with TGF-β receptors^30,49^). The activity of the epitope-tagged ENG was shown earlier^42,50^, and the activity of the tagged VEGFR2 constructs, measured following transfection of HEK293T cells that do not express VEGFR2^51^, is depicted in Supplementary Fig. 1. The activity of the epitope-tagged NRP1 is shown by its ability to enhance VEGF-A-mediated signaling and sprouting. The receptors at the plasma membrane were labeled with monovalent Fab’ fragments (anti-tag followed by a fluorescent secondary Fab’) and subjected to FRAP studies by a Gaussian-spot laser beam (see Methods). Representative FRAP curves for each receptor are shown in Fig. 1a-c, and the average values obtained from multiple FRAP measurements are given in Fig. 1d, e. All three receptors were laterally mobile, exhibiting lateral diffusion coefficients (*D*) and mobile fractions (*R*_f_) characteristic of transmembrane receptors (*D* from 2.0 to 3.6 × 10^-2^ μm^2^/s; *R*_f_ = 60–70%). These values are in the same range reported earlier in the same cells for ENG^30,39^. The *R*_f_ values suggest that there is also an immobile fraction, typical of transmembrane receptors, most likely due to interactions of the receptors with membrane-associated structures which are immobile on the FRAP timescale. Such mobility-restricting interactions were shown to occur with the membrane-underlying cytoskeleton, the extracellular matrix, and structures such as clathrin-coated pits^52–57^.

To measure the extent and mode (stable or transient) of complex formation between the various receptors situated at the plasma membrane of live cells, we employed patch/FRAP^43,47,58^. In this method (see Methods; for a schematic description, see^59^), two receptors with distinct extracellular epitope tags are coexpressed. One of them is patched and laterally immobilized by IgG crosslinking, while the other is labeled exclusively by monovalent fluorescent Fab’ fragments. The effects of immobilizing the first receptor on the lateral diffusion (*D* and *R*_f_) of the Fab’-labeled receptor are then measured by FRAP. Complex lifetimes longer than the characteristic FRAP times (i.e., stable interactions) reduce *R*_f_ with no effect on *D*, since bleached Fab’-labeled receptors do not undergo appreciable dissociation from the clusters of the crosslinked receptor during the FRAP measurement. Conversely, in the case of transient complexes (complex lifetimes shorter than the characteristic FRAP time), each Fab’-labeled receptor would undergo several dissociation/association cycles from the crosslinked receptor clusters during the FRAP measurement, reducing *D* without altering *R*_f_^43,47,58^. Using this method, we have demonstrated the formation of both stable and transient receptor complexes on the FRAP time scale; thus, stable interactions were found between the type II and type I (activin-like kinase 5; ALK5) TGF-β receptors^43^ or type II activin receptors and ALK4 or ALK1^47^, while type II bone morphogenetic protein (BMP) receptors exhibited transient interactions with their type I counterparts ALK3 or ALK6^45^.

**Fig. 2:**
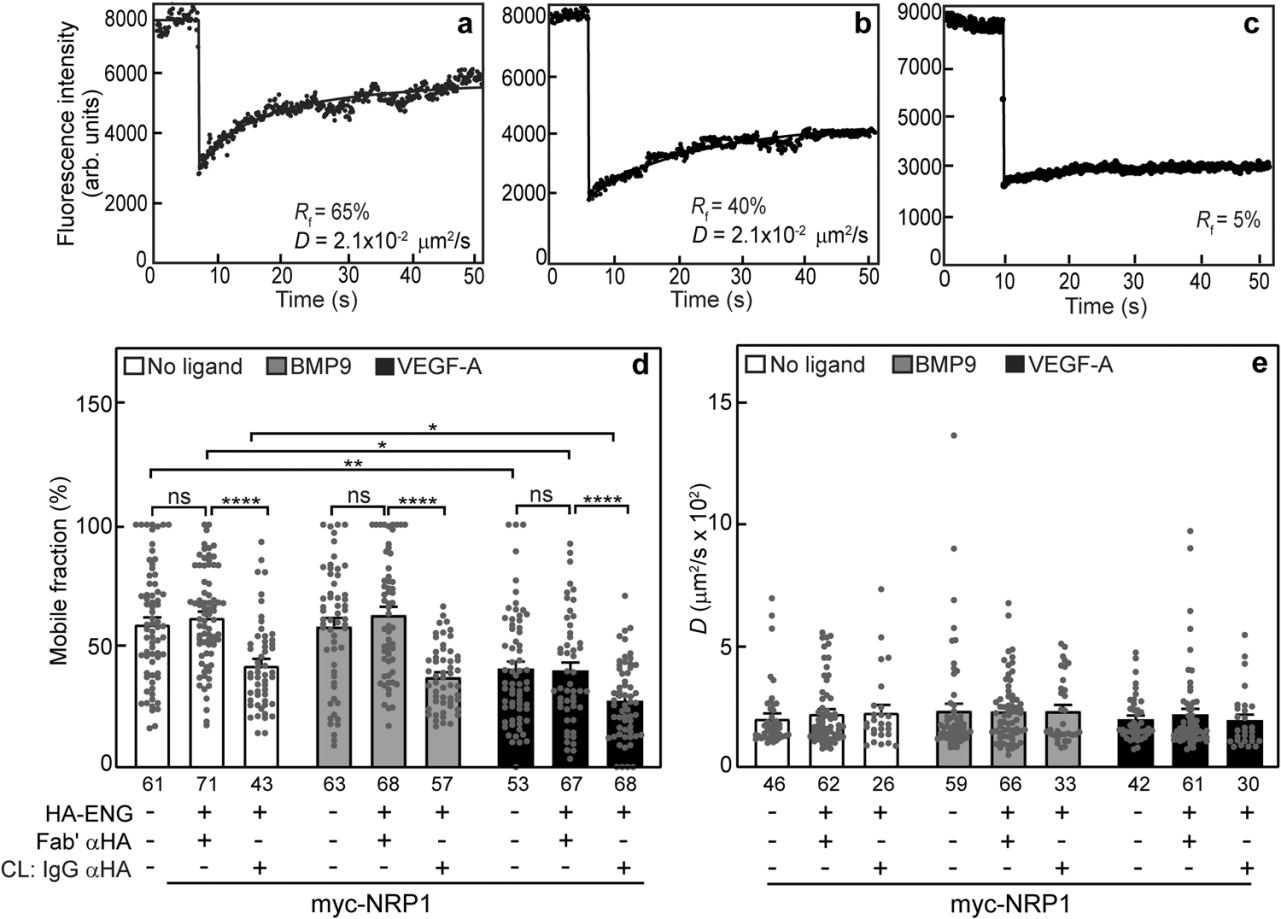
ENG and NRP1 form VEGF-A-sensitive heteromeric complexes. COS7 cells were co-transfected with vectors encoding myc-NRP1 alone or together with HA-ENG or empty vector (control). After 24 h, live cells were subjected to the IgG crosslinking (CL) protocol (see Methods), resulting in HA-ENG patched and labeled by Alexa 488-GαR IgG (designated CL: IgG αHA), and myc-NRP1 labeled exclusively by monovalent Fab’ (with Alexa 546-GαM Fab’ secondary antibody). In control experiments without crosslinking, HA-ENG was labeled by Fab’ instead of IgGs. Where indicated, ligand (5 ng/ml BMP9, or 50 ng/ml VEGF-A) was added at the last fluorescent labeling step for the FRAP experiment, and maintained at later steps. FRAP studies were conducted as in Fig. 1. Representative FRAP curves of myc-NRP1 coexpressed with uncrosslinked (Fab’-labeled) HA-ENG (**a**), after IgG-mediated CL HA-ENG (**b**), and of HA-ENG immobilized by IgG CL (**c**). Typical curves for the singly-expressed, Fab’-labeled receptors are given in Fig. 1. Average *R*_*f*_ (**d**) and *D* values (**e**) of the effect of HA-ENG coexpression and IgG-mediated immobilization on the lateral diffusion of myc-NRP1. Bars, mean ± SEM. The number of measurements (each conducted on a different cell) is shown under each bar. Some of these numbers are lower in the *D* panels, since only *R*_*f*_ can be extracted from FRAP curves yielding less than 20% recovery. Asterisks indicate significant differences between the *R*_*f*_ values of the pairs indicated by brackets (*p < 0.05; **p < 0.01; ****p < 10^−4^; one-way ANOVA and Bonferroni post-hoc test. ns = not significant). A similar analysis of the *D* values showed no significant differences.

To study the interactions between ENG and NRP1, cells were transfected by vectors encoding myc-NRP1 and HA-ENG (alone or together) under conditions yielding similar cell-surface expression levels (Methods). The effects of coexpressing HA-ENG (without or with its crosslinking by IgG) on the lateral diffusion of myc-NRP1, as well as the effects of ligands that bind to NRP1 (VEGF-A at 50 ng/ml, chosen based on former studies on VEGF-A-mediated signaling in ECs, where 25−100 ng/ml were used^42,60,61^) or ENG (BMP9 at 5 ng/ml, which was shown by us to produce the maximal response in the MEEC cell lines^62^), are depicted in Fig. 2. Typical FRAP curves of myc-NRP1 coexpressed with HA-ENG before and after IgG-crosslinking of the latter (Fig. 2a, b) are shown along with the immobilization of crosslinked HA-ENG (Fig. 2c). The average mean ± SEM of multiple measurements per condition are shown in Fig. 2d, e. Coexpression with HA-ENG without crosslinking did not alter significantly *R*_f_ and *D* of myc-NRP1, showing that binding of HA-ENG to myc-NRP1, direct or in a larger complex, has no effect on the lateral diffusion of myc-NRP1 as long as HA-ENG is not immobilized by crosslinking (Fig. 2d, e; compare the two leftmost bars in each group). However, IgG-mediated immobilization of HA-ENG led to a significant reduction in *R*_f_ of myc-NRP1 without alteration in its *D* value (Fig. 2d, e). Such an effect characterizes stable interactions between the HA- and myc-tagged receptors on the FRAP timescale^30,43,47^. Upon immobilization of HA-ENG, *R*_f_ of myc-NRP1 was reduced from 64 to 39%, suggesting that nearly 40% [(64-39)/64, yielding 39%] of the NRP1 molecules are in stable complexes with ENG already prior to ligand binding. Of note, VEGF-A reduced *R*_f_ of myc-NRP1 either singly-expressed or coexpressed with HA-ENG (without or with crosslinking) (Fig. 2d), with no effect on *D* (Fig. 2e). The effect of VEGF-A on singly-expressed myc-NRP1 indicates that it enhances NRP1 binding to cellular structures that are laterally immobile on the FRAP timescale^52–54,57^. In cells coexpressing HA-ENG/myc-NRP1, incubation with VEGF-A (but not BMP9) followed by IgG crosslinking of HA-ENG further reduced *R*_f_ of myc-NRP1 (from 39 to 26%; Fig. 2d), leaving *D* unaffected (Fig. 2e). This indicates that VEGF-A enhances the interactions between ENG and NRP1.

To test for the specificity of the inhibition of the lateral diffusion of *e.g*. myc-NRP1 by crosslinking HA-ENG, we conducted control experiments where HA-ENG was replaced by HA-tagged activin receptor type 2B (ACVR2B), an unrelated transmembrane receptor. As shown in Supplementary Fig. 2, IgG crosslinking of HA-ACVR2B had no significant effect on either the *R*_f_ or *D* values characterizing the lateral diffusion of myc-NRP1.

**Fig. 3:**
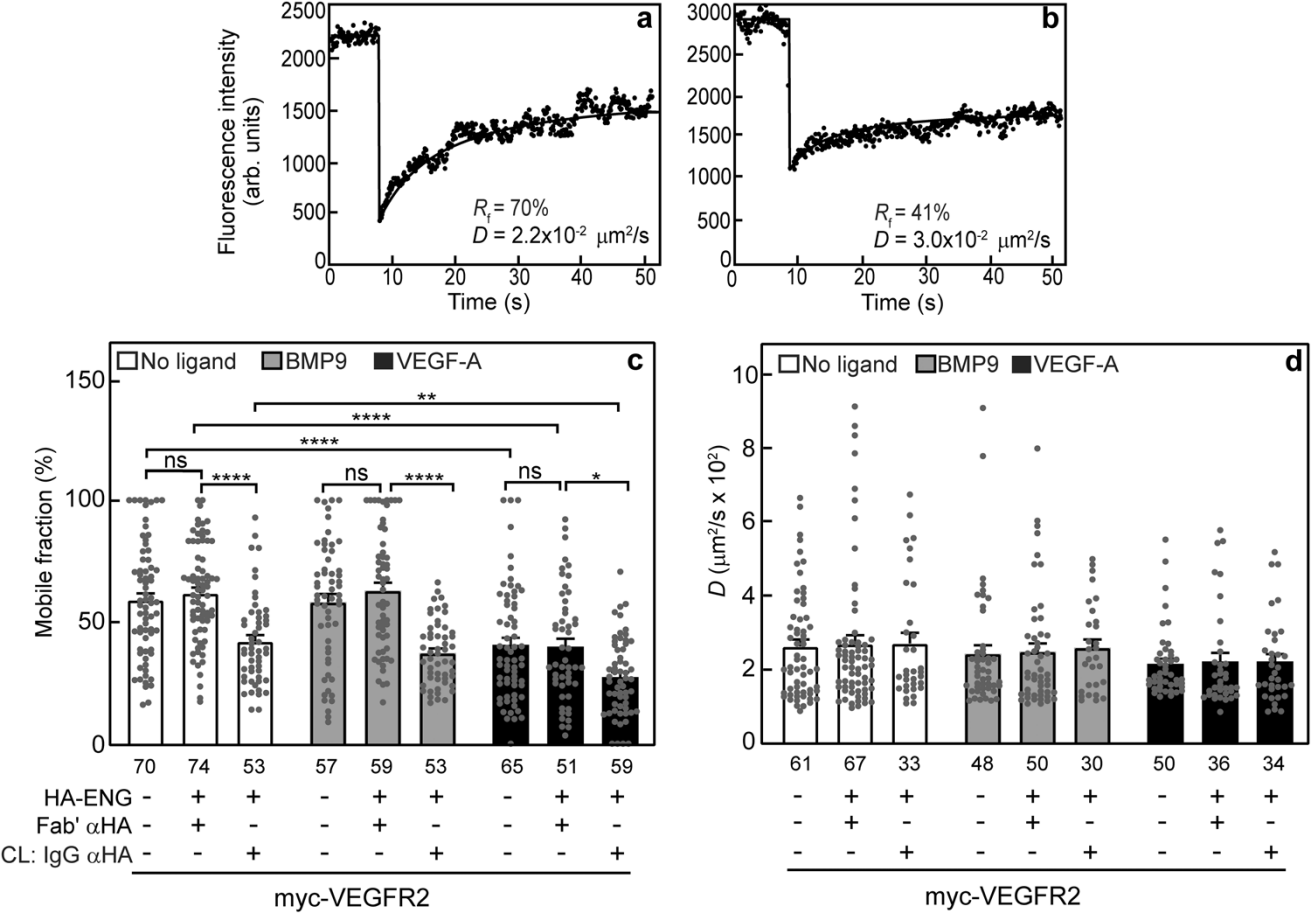
ENG and VEGFR2 form stable complexes which are enhanced by VEGF-A. COS7 cells were co-transfected with expression vectors encoding myc-VEGFR2 alone or together with HA-ENG (or empty vector) as in Fig. 2. Where shown, HA-ENG was immobilized by IgG-crosslinking as in Fig. 2. The lateral mobility of Fab’-labeled myc-VEGFR2 was measured by FRAP. Where indicated, BMP9 (5 ng/ml) or VEGF-A (50 ng/ml) were added during the last fluorescent labeling step for the FRAP experiment, and maintained thereafter. Representative FRAP curves of the lateral diffusion of myc-VEGFR2 coexpressed with uncrosslinked (**a**) or IgG-immobilized HA-ENG (**b**). Average *R*_f_ (**c**) and *D* values (**d**) showing the effect of coexpression and immobilization of HA-ENG (IgG crosslinking; CL) on the lateral diffusion of myc-VEGFR2. The bars depict the average values (mean ± SEM); the number of measurements (each conducted on a different cell) is shown under each bar. Asterisks indicate significant differences between the *R*_f_ values of the pairs indicated by brackets (*p < 0.05; **p < 0.01; ****p < 10^−4^; one-way ANOVA and Bonferroni post-hoc test. ns = not significant). A similar analysis of the *D* values showed no significant differences.

Representative FRAP curves of myc-VEGFR2 coexpressed with uncrosslinked or IgG-immobilized HA-ENG are depicted in Fig. 3a, b, respectively. The effects of IgG-mediated crosslinking of HA-ENG on the lateral diffusion of myc-VEGFR2 followed the same pattern as ENG/NRP1 interactions, indicating stable complex formation between HA-ENG and myc-VEGFR2 (Fig. 3c, d). Immobilization of HA-ENG significantly reduced *R*_f_ of Fab’-labeled myc-VEGFR2 (from 62 to 42%) without altering the *D* value (Fig. 3c, d). This suggests that ~32% of the myc-VEGFR2 molecules at the cell surface are in stable complexes with HA-ENG. The reduction in *R*_f_ of myc-VEGFR2 did not occur upon coexpression with uncrosslinked HA-ENG, demonstrating that without immobilization of the surface HA-ENG molecules, their interaction with myc-NRP1 does not affect its lateral mobility (Fig. 3c, d; compare the two leftmost bars in each group). Of note, similar results were obtained when the experiment was performed in reversed order, i.e., myc-ENG was immobilized by IgG crosslinking, and the effect on the lateral diffusion of coexpressed HA-VEGFR2 was measured (Supplementary Fig. 3). Here, immobilization of myc-ENG reduced *R*_f_ of Fab’-labeled HA-VEGFR2 from 52% to 36%, indicating that 31% of HA-VEGFR2 are in stable complexes with myc-ENG, a result essentially identical to that observed for the reverse experiment. The similarity to the interactions of ENG with NRP1 was seen also in the effects mediated by ligands. VEGF-A, but not BMP9 (an ENG ligand), reduced *R*_f_ (with no effect on *D*) of myc-VEGFR2 whether singly-expressed or coexpressed with HA-ENG (without or with crosslinking) (Fig. 3c, d). The reduced *R*_f_ of singly-expressed myc-VEGFR2 upon binding VEGF-A suggests that VEGF-A enhances its interactions with other membrane associated proteins and/or cellular structures with restricted mobility. Of note, VEGF-A binding enhances the complex between ENG and VEGFR2, as indicated by the increased reduction in *R*_f_ of myc-VEGFR2 coexpressed with HA-ENG upon immobilization of the latter (*R*_f_ reduced from 42 to 27%; Fig. 3c).

**Fig. 4:**
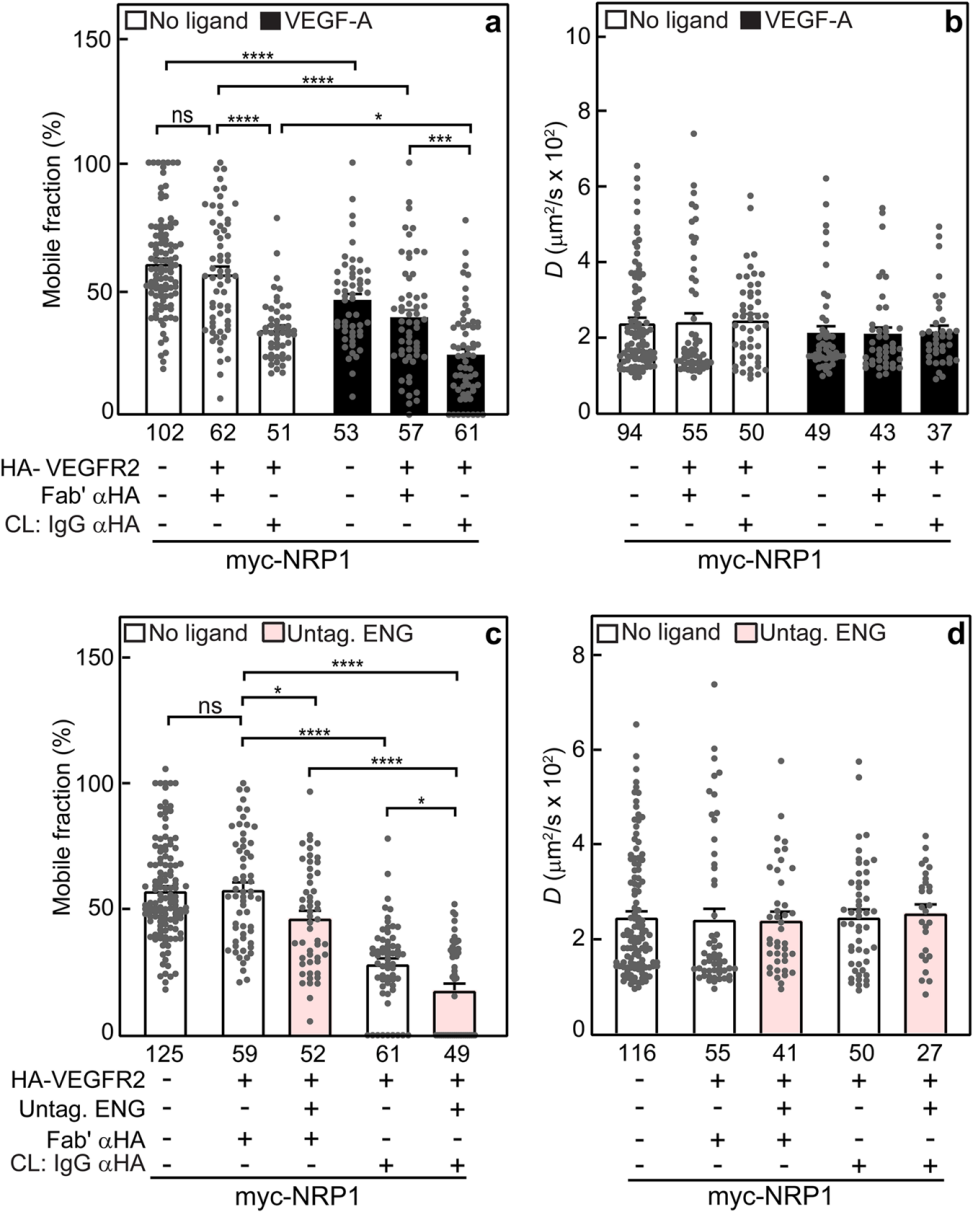
ENG enhances complex formation between NRP1 and VEGFR2. To study whether ENG can participate in complexes with both NRP1 and VEGFR2, we conducted patch/FRAP studies on COS7 cells expressing myc-NRP1 with HA-VEGFR2 (or empty vector) as described in Fig. 3. Where indicated, HA-VEGFR2 was immobilized by IgG as in Fig. 2. The lateral mobility of Fab’-labeled myc-NRP1 was measured by FRAP. FRAP studies on NRP1/VEGFR2 complex formation, depicting the average *R*_f_ (**a**) and *D* values (**b**) in the absence or presence of VEGF-A. **c, d** Studies on the modulation of NRP1/VEGFR2 interactions by ENG. The average *R*_f_ (**c**) and *D* values (**d**) of myc-NRP1 were measured. Where indicated, untagged ENG was coexpressed along with the tagged receptors (myc-NRP1 and HA-VEGFR2), followed by IgG crosslinking (CL) of HA-VEGFR2. Bars are mean ± SEM; the number of measurements (on different cells) appears under each bar. The reduction in *R*_f_ of myc-NRP1 was significantly reduced upon IgG CL of HA-VEGFR2, an effect enhanced by VEGF-A, either in the absence (**a**) or presence (c) of untagged ENG. Coexpression with ENG by itself (without IgG CL of HA-VEGFR2) already induced a mild reduction in *R*_f_ of myc-NRP1, which was significantly more pronounced upon immobilization of HA-VEGFR2 (compare **a**, **c**). These results demonstrate that the presence of ENG enhances NRP1/VEGFR2 interactions, and may serve as a bridge to form a ternary complex. Asterisks indicate significant differences between the *R*_f_ values of the pairs indicated by brackets (*p < 0.05; ***p < 10^−3^; ****p < 10^−4^; one-way ANOVA and Bonferroni post-hoc test. ns = not significant). A similar analysis of the *D* values showed no significant differences in all cases.

Next, we investigated VEGFR2/NRP1 complex formation (Fig. 4a, b) and its modulation by ENG (Fig. 4c, d). To measure VEGFR2/NRP1 interactions, we performed patch/FRAP experiments on cells transfected with myc-NRP1 and/or HA-VEGFR2, measuring the effects of coexpressing HA-VEGFR2 (without or with IgG crosslinking) on the lateral diffusion of myc-NRP1 to determine the formation of mutual complexes and their potential modulation by VEGF-A. As shown in Fig. 4a, b, expression of HA-VEGFR2 without crosslinking had no effect on *R*_f_ or *D* of myc-NRP1, suggesting that coexpression with HA-VEGFR2 without its immobilization does not affect the lateral diffusion of myc-NRP1. IgG crosslinking of HA-VEGFR2 resulted in a strong reduction in *R*_f_ of myc-NRP1 (from 56 to 34%), leaving *D* unaltered. This is indicative of stable complex formation between ~39% of the population of these receptors. VEGF-A markedly reduced further the *R*_f_ values of myc-NRP1, expressed either alone or together with HA-VEGFR2, following the same pattern measured for the interactions between ENG/NRP1 (Fig. 2d, e) and ENG/VEGFR2 (Fig. 3c, d). The reduction in *R*_f_ of singly-expressed myc-NRP1 was similar to that observed in Fig. 2 under the same conditions, supporting the notion that VEGF-A-mediates enhanced interactions of NRP1 with mobility-restricted proteins or cellular structures. The further reduction induced by VEGF-A in *R*_f_ of myc-NRP1 upon immobilization of coexpressed HA-VEGFR2 (from 34 to 24%; Fig. 4a) suggests that the ligand enhances the interactions between VEGFR2 and NRP1, as expected. It should be noted that while the results in Fig. 4a indicate that NRP1 and VEGFR2 interact to some degree without VEGF-A, these complexes are likely inactive in the absence of ligand; VEGF-A binding may then induce activation of the pre-existing complexes, and concomitantly enhance the formation of VEGFR2/NRP1 complexes.

Based on the interactions of ENG with both NRP1 and VEGFR2, it was of interest to explore whether the latter two receptors could bind simultaneously to ENG. To this end, we conducted patch/FRAP studies on the effects of overexpressing untagged ENG on the interactions between HA-VEGFR2 and myc-NRP1 (Fig. 4c, d). Cells were transfected with myc-NRP1 alone, together with HA-VEGFR2, or together with both HA-VEGFR2 and untagged ENG. HA-VEGFR2 was subjected (or not; control) to IgG crosslinking, and all samples were taken for patch/FRAP studies to determine VEGFR2/NRP1 complex formation in the absence or presence of untagged ENG. Overexpression of untagged, free (uncrosslinked) ENG induced a reduction in *R*_f_ of myc-NRP1 coexpressed with HA-VEGFR2 even without IgG crosslinking of HA-VEGFR2 (from 58 to 47%; Fig. 4c). An important control is provided by the studies on the effects of uncrosslinked HA-ENG on either myc-NRP1 or myc-VEGFR2 mobility, which showed that in the absence of HA-ENG crosslinking, ENG has no effect on the lateral diffusion of NRP1 or VEGFR2 (Figs. 2d, e and 3c, d; compare the two leftmost bars in each panel). Immobilization of HA-VEGFR2 (without overexpressing ENG) reduced *R*_f_ of myc-NRP1 (from 56% to 28%). Of note, this reduction became significantly stronger (down to 18%, suggesting [56-18/56] = 68% in mutual complexes) upon coexpression of untagged, uncrosslinked ENG as a third component (Fig. 4c). The *D* value of myc-NRP1 was unaffected in all cases, suggesting that the interactions are stable. Because untagged ENG is not crosslinked in these experiments, and uncrosslinked ENG does not affect the diffusion of either NRP1 or VEGFR2, these findings indicate that overexpressed untagged ENG enhances the interactions between VEGFR2 and NRP1 by forming mutual complexes with them. This brings up the possibility that ENG may serve as a bridge between VEGFR2 and NRP1, which bind to it at different sites. In such a case, NRP1 is expected not to compete with VEGFR2 for binding to ENG. As shown in Supplementary Fig. 4, this expectation is met, as overexpression of untagged NRP1 has no effect on the ability of immobilized HA-ENG to reduce *R*_f_ of myc-VEGFR2.

### ENG and NRP1 enhance VEGF-A-mediated phosphorylation of VEGFR2 and Erk1/2 in murine embryonic endothelial cells (MEECs)

To investigate whether the formation of complexes between the three receptors correlates with signaling, we studied the effects of ENG on VEGF-A-induced formation of pVEGFR2 and pErk1/2 in MEEC^+/+^ (expressing ENG) *vs*. MEEC^-/-^ (ENG-null) endothelial cells^42,48^. The role of complex formation with NRP1 was investigated by overexpressing NRP1 or its knockdown by siRNA in these cell lines. Since these studies depend on the endogenous receptors in the MEEC lines, we first determined the endogenous mRNA and protein levels of the three receptors in MEEC^+/+^ and MEEC^-/-^ cells. The mRNA levels of *ENG*, *NRP1* and *VEGFR2* determined by RT-qPCR are shown in Supplementary Fig. 5a. *ENG* mRNA was expressed only in MEEC^+/+^ cells, while *NRP1* and *VEGFR2* mRNAs were expressed in both cell lines, albeit to a lower extent (especially *NRP1*) in the MEEC^-/-^ cells. However, determination of the levels of the latter two proteins by Western blotting (Supplementary Fig. 5b−d) indicated similar expression levels for VEGFR2 in the two MEEC lines, and a 2-fold difference for NRP1. Next, to select the optimal stimulation time with VEGF-A, we measured the time course of VEGF-A-mediated formation of pVEGFR2 and pErk1/2 in MEEC^+/+^ and MEEC^-/-^ cells. After serum starvation and stimulation with VEGF-A (50 ng/ml) for the indicated times, pVEGFR2, total VEGFR2 (tVEGFR2), pErk1/2 and total Erk1/2 (tErk1/2) were measured by Western blotting (Supplementary Fig. 6). In both cell lines, the strongest stimulation was obtained at 5 min, which was selected for further experiments. In accord with former reports^42^, signaling to both pVEGFR2 and pErk1/2 relative to the total levels of these proteins was higher in MEEC^+/+^ than in MEEC^-/-^, suggesting that ENG enhances signaling by VEGF-A in these cells.

**Fig. 5:**
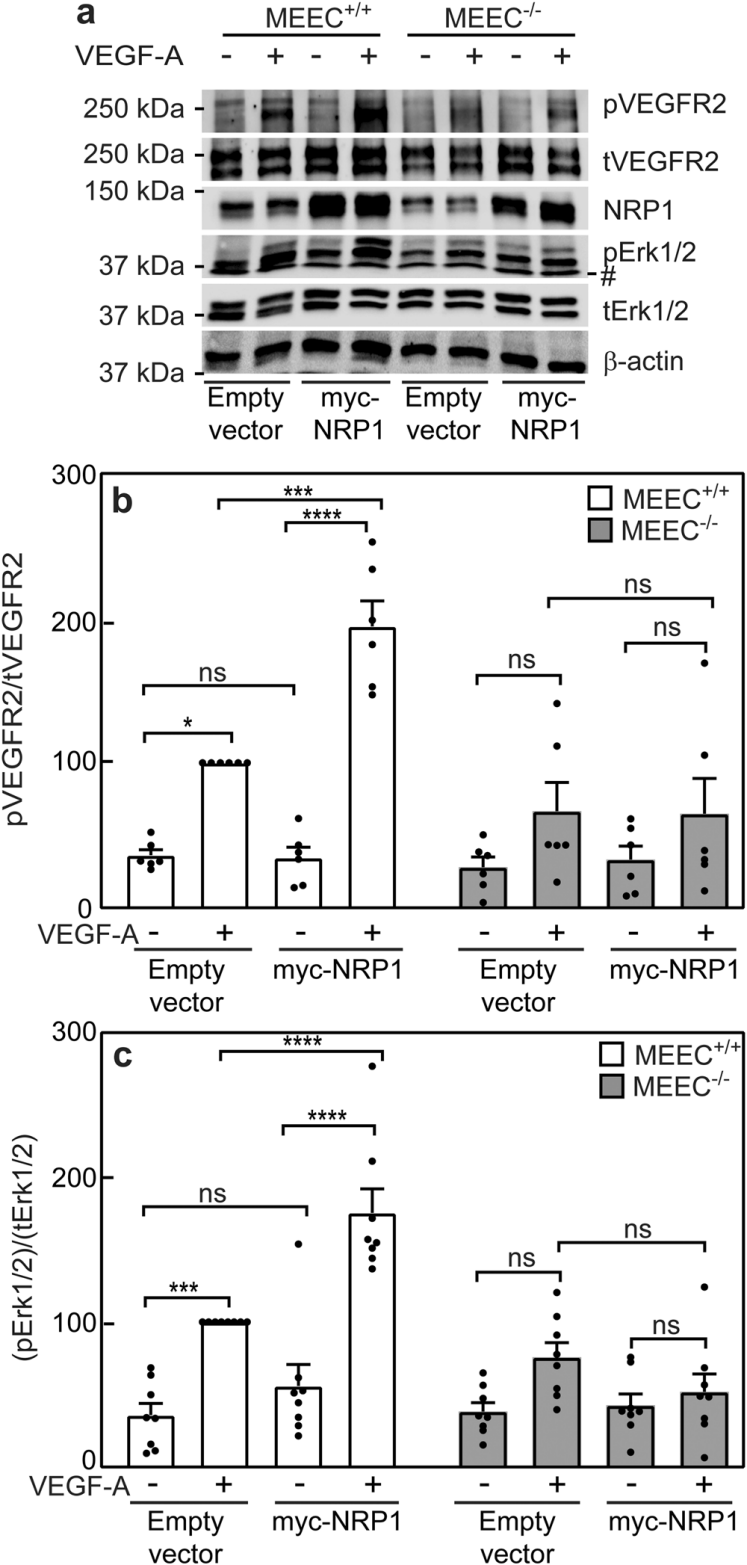
NRP1 overexpression in the presence of ENG enhances VEGF-A-mediated signaling in MEECs. MEEC^+/+^ and MEEC^-/-^ cells were transfected with myc-NRP1 or empty vector (control) as described under Methods. After 24 h, they were serum starved (30 min) followed by stimulation (5 min) with 50 ng/ml VEGF-A. Cell lysates were subjected to SDS-PAGE and immunoblotted for pVEGFR2, tVEGFR2, NRP1, pErk1/2, tErk1/2 and β-actin. **a** Representative immunoblot. Quantification of the effect of NRP1 overexpression on VEGF-A signaling to pVEGFR2 (**b**) or to pErk1/2 (**c**). The bands were visualized by ECL and quantified by densitometry. Data are mean ± SEM of 6−8 independent experiments. The values obtained for VEGF-A-stimulated MEEC^+/+^ transfected with empty vector were taken as 100%. Asterisks indicate significant differences between pairs of MEEC^+/+^ or MEEC^-/-^ cells with or without myc-NRP1 overexpression (one-way ANOVA and Bonferroni post-hoc test; *p < 0.05; ***p < 10^-3^; ****p < 10^-4^). ns = not significant. The band marked by # in the representative blot (**a**) is non specific.

To explore whether NRP1 modulates VEGF-A signaling, MEEC^+/+^ and MEEC^-/-^ cells were transfected with myc-NRP1. After 24 h, the cells were subjected to the signaling assays as described under Methods and in Supplementary Fig. 6. As shown in Fig. 5, overexpression of myc-NRP1 increased VEGF-A-mediated formation of pVEGFR2 and pErk1/2 in MEEC^+/+^ but not in MEEC^-/-^ cells, indicating that ENG is required for enhancement of VEGF-A signaling by NRP1. However, because MEEC^-/-^ cells also express less NRP1 than MEEC^+/+^, part of their failure to respond to VEGF-A could be due to the lower NRP1 level. To test this, we overexpressed ENG and/or NRP1 on the same cellular background. MEEC^-/-^ cells were transfected either with HA-ENG alone or together with myc-NRP1, and the effects on VEGF-A-mediated signaling to pVEGFR2 and pErk1/2 were measured (Supplementary Fig. 7). The results demonstrate that expression of ENG alone induced a mild increase in VEGF-A-mediated signaling in MEEC^-/-^ cells, in line with the notion that ENG is necessary for these responses. Coexpression of NRP1 together with ENG strongly promoted VEGF-A signaling in the MEEC^-/-^ cells, suggesting that a high expression level of NRP1 enhances VEGF-A-mediated signaling in these cells. Together with the finding that overexpression of myc-NRP1 alone in MEEC^-/-^ cells (Fig. 5; conditions under which there is still no ENG, but the NRP1 level is high) is insufficient to promote VEGF-A signaling, we conclude that ENG is required to induce the above signaling responses, but optimal VEGF-A-mediated phosphorylation of VEGFR2 and Erk1/2 requires in addition a significant level of NRP1.

**Fig. 6:**
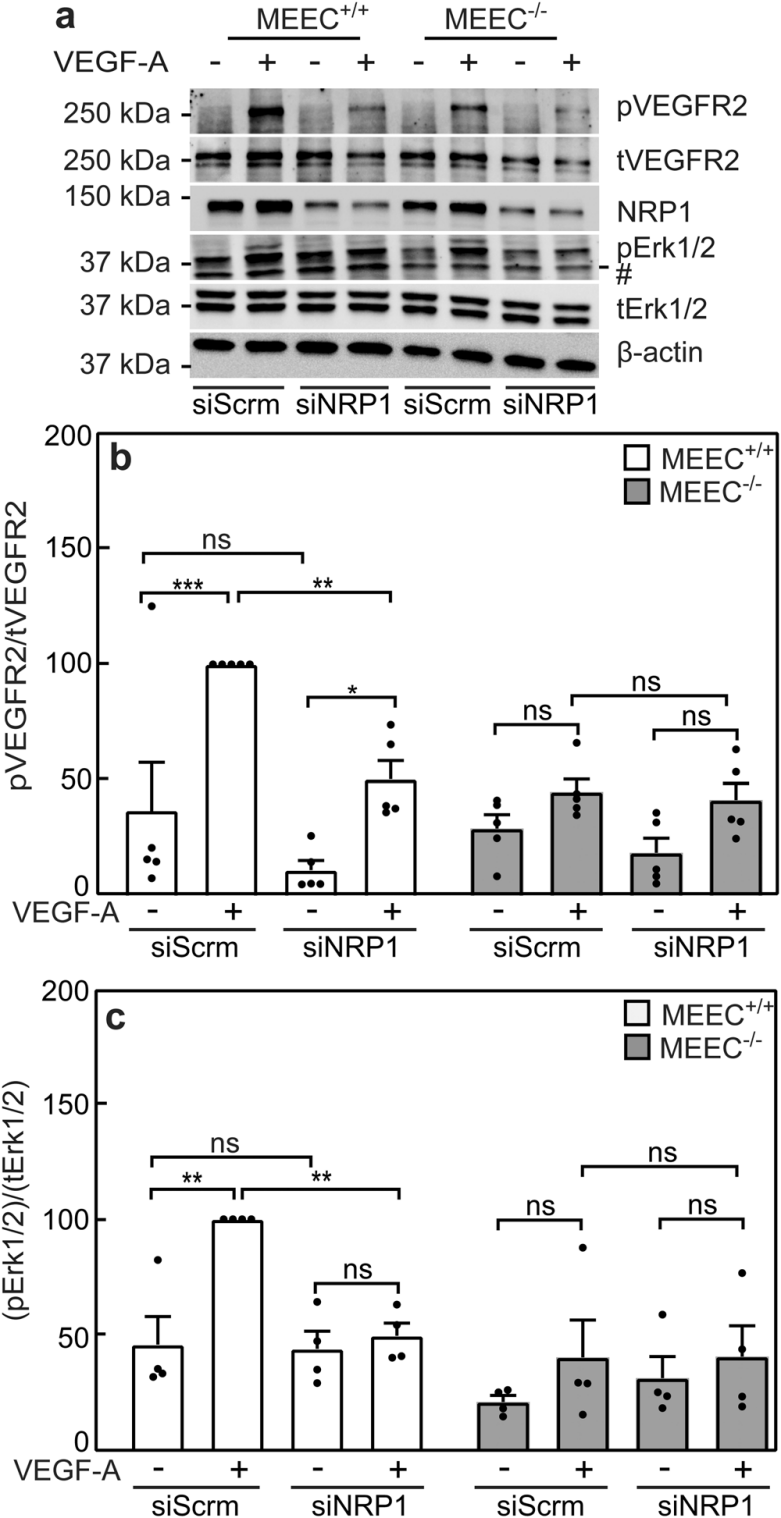
*NRP1* knockdown inhibits VEGF-A signaling in MEECs expressing ENG. MEEC^+/+^ and MEEC^-/-^ cells were transfected with siRNA to *NRP1* or scrambled siRNA (siScrm; control). After 48 h, conditions which effectively downregulated the mRNA levels of *NRP1* (Supplementary Fig. 8), they were taken for signaling studies conducted as described in Fig. 5. **a** A representative immunoblot. Quantification of the effect of si*NRP1* on VEGF-A signaling to pVEGFR2 (**b**) or to pErk1/2 (**c**). The bands were visualized by ECL and quantified by densitometry. Data are mean ± SEM of 4−5 independent experiments. The values obtained for VEGF-A-stimulated MEEC^+/+^ transfected with siScrm were taken as 100%. Asterisks indicate significant differences between pairs of MEEC^+/+^ or MEEC^-/-^ cells with or without si*NRP1* treatment (one-way ANOVA and Bonferroni post-hoc test; *p < 0.05; **p < 0.01; ***p < 10^-3^). ns = not significant. The band marked by # in the representative blot (**a**) is non specific.

To complement these studies, we examined the effects of reducing the level of *NRP1* by siRNA on VEGF-A signaling in the two MEEC cell lines. As shown in Fig. 6, siRNA to *NRP1* (si*NRP1*), whose effectiveness is shown in Supplementary Fig. 8, reduced significantly the ability of VEGF-A to induce pVEGFR2 and pErk1/2 in MEEC^+/+^ cells, while in the MEEC^-/-^ cells there was no effect as their signaling is lost due to the lack of ENG and the low level of NRP1. This finding is in line with the above conclusions, and is in accord with our demonstration of the formation of a triple complex containing VEGFR2, NRP1 and ENG (Fig. 4c).

### ENG and NRP1 affect VEGF-A-induced sprouting of MEECs

**Fig. 7:**
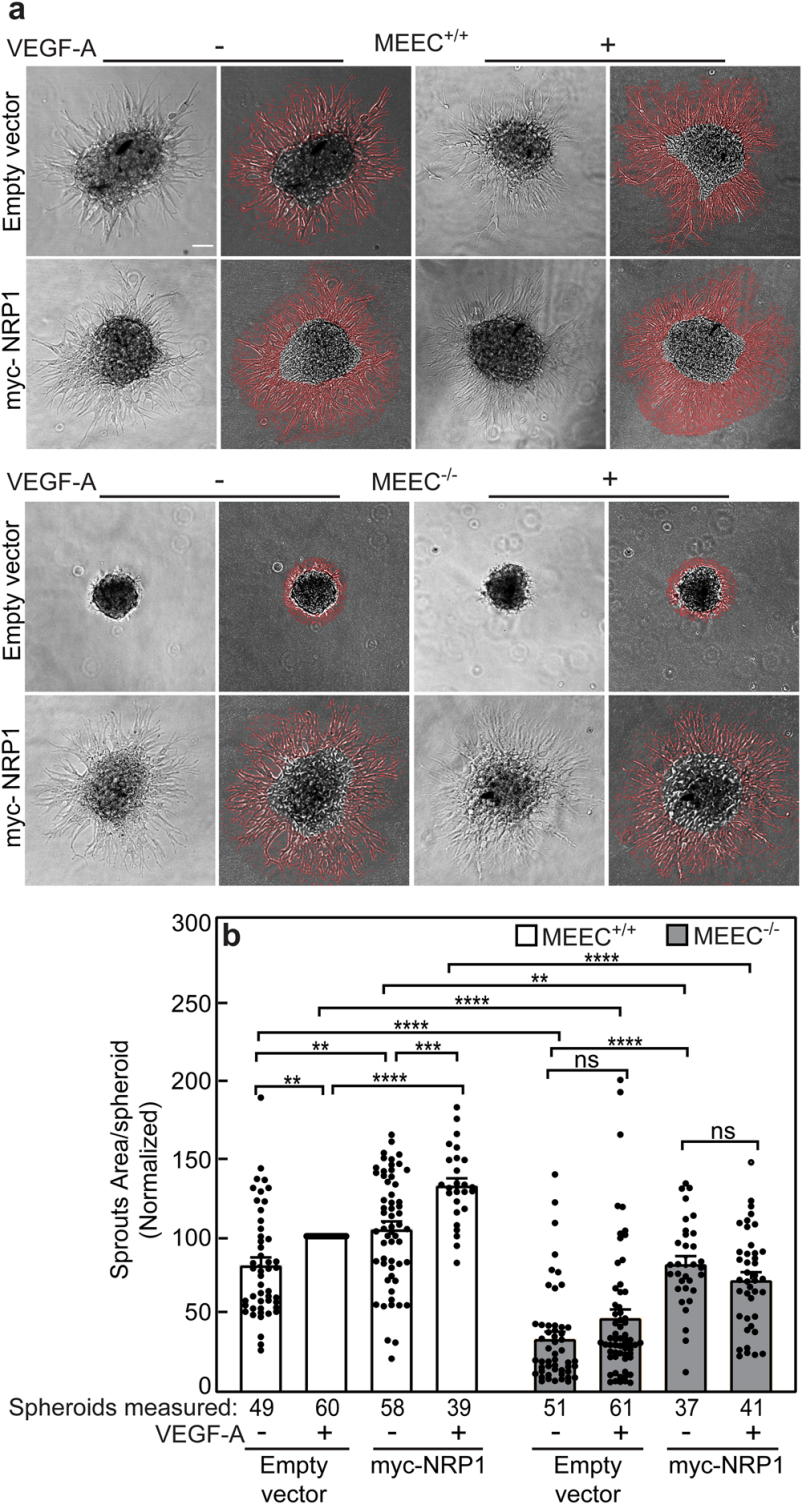
NRP1 overexpression enhances vascular sprouting in MEECs. MEEC^+/+^ and MEEC^-/-^ cells were transfected with myc-NRP1 or empty vector as in Fig. 5. At 24 h post-transfection, they were lifted in full medium and cultured as hanging drops (2 × 10^5^ cells/drop; 24 h). The spheroids were collected, and allowed to sprout on growth factor-reduced Matrigel with or without VEGF-A (100 ng/ml) for another 24 h (Methods). Phase-contrast images were taken with 10x magnification, and Image Pro Plus was used to outline the sprouts and quantify the area covered by them for each spheroid (Methods). **a** Typical images of spheroids analysed for sprouts area. Each original phase contrast image (left) is shown alongside the image with red-outlined sprouts. Scale bar, 100 µm. **b** Quantification of the sprouting experiments. Sprouting was enhanced by overexpression of NRP1 either in the absence (MEEC^-/-^) or presence (MEEC^+/+^) of ENG, while VEGF-A-mediated sprouting required ENG. Data are mean ± SEM of *n* = 37−61 spheroids per condition (the number of spheroids measured are indicated under each bar) from 5 independent experiments. The area of the sprouts in VEGF-A-stimulated MEEC^+/+^ was normalized to 100%, and the sprouts area under all other conditions was calculated relative to this value. **p < 0.01; ***p < 10^-3^; ****p < 10^-4^ (one-way ANOVA and Bonferroni post-hoc test). ns = not significant.

Sprouting of ECs is an important process in the formation of blood vessels. We therefore investigated the effects of ENG and NRP1 on VEGF-A-mediated sprouting of MEEC^+/+^ and MEEC^-/-^. To this end, we employed a hanging drop sprouting assay (Methods) to measure the effects of overexpression or knock down of *NRP1* on VEGF-A-mediated sprouting of MEEC^+/+^ *vs*. MEEC^-/-^. In cells transfected with empty vector, MEEC^+/+^ exhibited more sprouting with larger spheroids than MEEC^-/-^, and VEGF-A significantly stimulated sprouting only in MEEC^+/+^, in line with a requirement for ENG^42^ (Fig. 7a, b). Overexpression of NRP1 mildly increased sprouting in unstimulated MEEC^+/+^ cells. VEGF-A significantly enhanced sprouting in MEEC^+/+^ cells whether or not transfected with NRP1, while myc-NRP1 overexpressing cells exhibited significantly higher sprouting than cells transfected with empty vector (Fig. 7a, b). In accord with the signaling studies, U0126, an inhibitor of Mitogen-activated protein kinase kinase 1 and 2 (MEK1/2), inhibited both the phosphorylation of Erk1/2 and the sprouting of MEEC^+/+^ cells either without or with NRP1 overexpression (Supplementary Fig. 9), in line with the reports on VEGF-A activation of MEK1/2 to phosphorylate Erk1/2^9,63^, and on the requirement of Erk1/2 activation for cell proliferation, migration and angiogenesis^64^. In MEEC^-/-^, NRP1 overexpression also increased sprouting (and spheroid size) in the absence of VEGF-A; however, the sprouting level of MEEC^-/-^ cells overexpressing NRP1 remained significantly lower than that in MEEC^+/+^ under the same conditions (Fig. 7a, b). Of note, even with overexpressed NRP1, MEEC^-/-^ sprouting remained insensitive to VEGF-A, in line with the absence of ENG in these cells. In view of the lower NRP1 expression in MEEC^-/-^ cells, we conducted sprouting experiments designed to validate the role of ENG and NRP1 in the same cellular background. MEEC^-/-^ cells were transfected with HA-ENG alone, or together with myc-NRP1. This was followed by studies on the effects of the transfected ENG and NRP1 on sprouting, either without or with VEGF-A. As depicted in Supplementary Fig. 10, sprouting of MEEC^-/-^ cells was enhanced by ENG overexpression already without ligand and exhibited a further mild increase upon VEGF-A stimulation. Of note, it was significantly increased upon co-transfection of myc-NRP1 together with HA-ENG, indicating that optimal sprouting response requires ENG and is enhanced by high levels of NRP1.

**Fig. 8:**
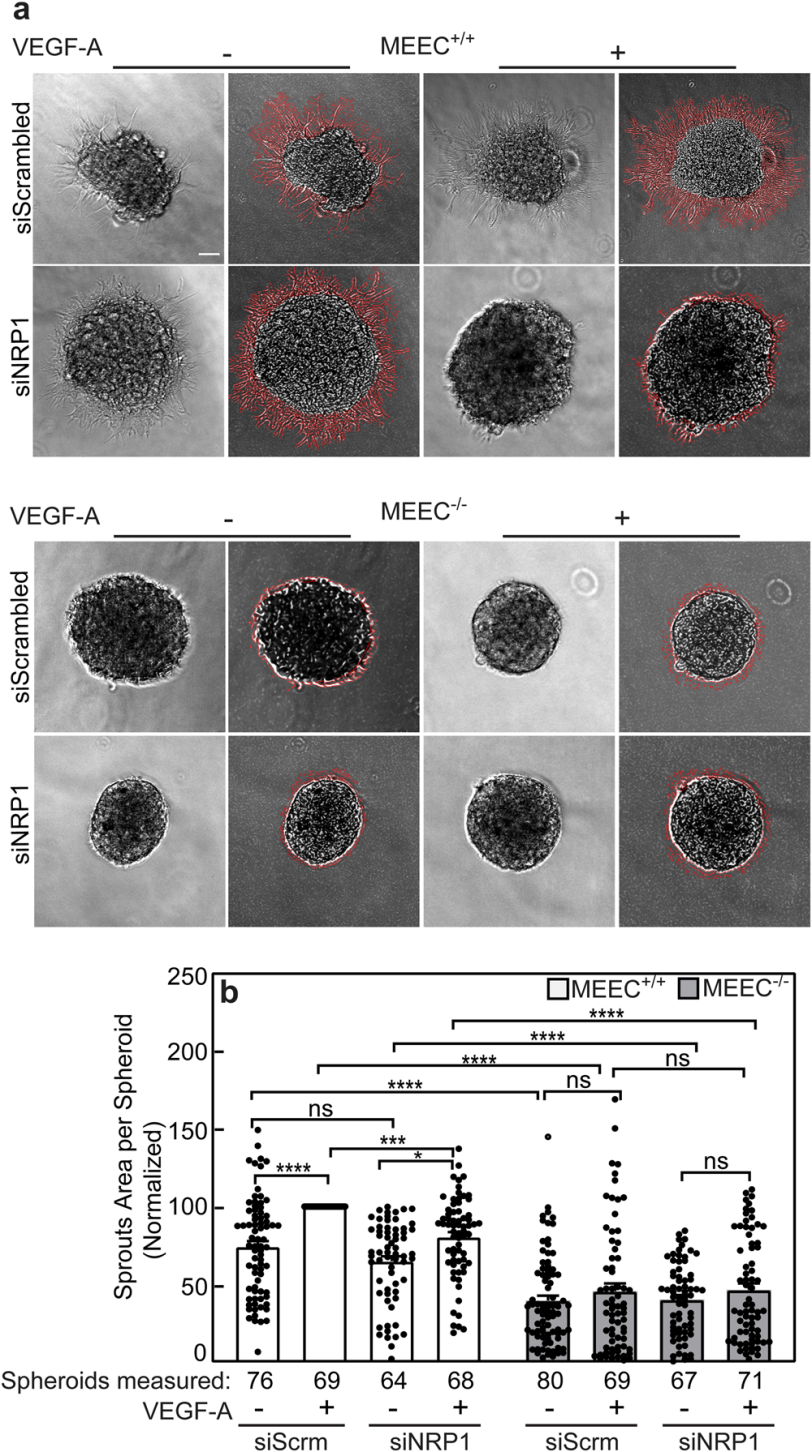
*NRP1* knockdown inhibits VEGF-A signaling in MEECs expressing ENG. MEEC^+/+^ and MEEC^-/-^ cells were transfected with siRNA to *NRP1* or scrambled siRNA (siScrm; control). After 24 h, they were taken for sprouting experiments based on the hanging drop assay as described in Fig. 7. **a** Typical images of spheroids analysed for sprouts area. The phase contrast images (left) are depicted together with the equivalent images with the outlined sprouts. Scale bar, 100 µm. **b** Quantification of the sprouting experiments. Silencing *NRP1* canceled the VEGF-A-mediated enhanced sprouting in the presence of ENG (MEEC^+/+^ cells). There was no effect on the ENG-null MEEC^-/-^ cells, where the normal level of sprouting is low to begin with. Data are mean ± SEM of *n* = 60−80 spheroids per condition (the number of spheroids measured is indicated under each bar) from 5 independent experiments. The area of the sprouts in VEGF-A-stimulated MEEC^+/+^ was normalized to 100%, and the sprouts area under all other conditions was calculated relative to this value. *p < 0.05; ****p < 10^-4^ (one-way ANOVA and Bonferroni post-hoc test). ns = not significant.

We further tested the effect of si*NRP1* on sprouting of MEEC^+/+^ and MEEC^-/-^ cells (Fig. 8). While VEGF-A still increased sprouting in si*NRP1*-treated MEEC^+/+^ cells, the sprouting level attained was significantly lower than in the siScrm control cells (Fig. 8a, b). On the other hand, MEEC^-/-^ cells remained VEGF-A-insensitive irrespective of the treatment with si*NRP1*. Taken together, these findings confirm the requirement of ENG for VEGF-A-mediated sprouting of MEEC cells at all levels of NRP1 expression (endogenous at higher (MEEC^+/+^) or lower (MEEC^-/-^) levels, overexpressed, or knocked down), and that NRP1 contributes to enhanced VEGF-A-induced sprouting with a dependence on its expression level.

## Discussion

VEGF-A is a major inducer of sprouting and angiogenesis of ECs^4^. It does so mainly *via* binding to and activation of VEGFRs, the most prominent of which is VEGFR2^5–7^. A co-receptor of VEGFR2 is NRP1, which also binds VEGF-A and modulates VEGFR2 signaling^8,9,12–14^. Moreover, ENG, which is highly expressed in ECs, was also reported to interact with VEGFR2 or NRP1, thus contributing to VEGF signaling and vascular sprouting^41,42^. These reports give rise to the hypothesis that interactions between ENG, VEGFR2 and/or NRP1 can regulate EC sprouting and angiogenesis. However, the formation and dynamics of complexes between these three proteins situated at the cell surface were not characterized. Here, we investigated these issues by biophysical studies on the interactions between ENG, NRP1 and VEGFR2, combined with studies of VEGF-A-induced signaling and biological outcome in ECs.

In order to enable the biophysical patch/FRAP experiments on the interactions between the three receptors, we initially characterized the lateral diffusion coefficients and mobile fractions of each of the singly-expressed epitope-tagged receptors. The *D* and *R*_f_ values obtained (Fig. 1d, e) were all in the range reported for other transmembrane proteins, including ENG^30,39,43,45,47,49^. We proceeded to characterize the interactions between myc-NRP1 and HA-ENG by coexpressing HA-ENG with myc-NRP1, and measuring the effects on the lateral diffusion of the latter. It should be noted that the FRAP studies were carried out at a lower temperature in order to inhibit endocytosis, and thus the interaction kinetics could vary at 37 °C, although the general tendency of receptor dynamics and interactions is retained^65,66^. While HA-ENG coexpression did not affect either *D* or *R*_f_ of myc-NRP1, immobilization of HA-ENG by IgG crosslinking significantly reduced *R*_f_ (but not *D*) of myc-NRP1 (Fig. 2d, e). This indicates stable complexes between the two receptors prior to VEGF-A binding^30,43,47^. The lack of effect on *D* reflects the weak dependence (logarithmic) of the lateral diffusion of transmembrane proteins on the membrane-embedded protein mass^67^. Interestingly, incubation with VEGF-A (but not BMP9) reduced *R*_f_ of singly-expressed myc-NRP1 (Fig. 2d), indicating that VEGF-A enhances NRP1 binding to other endogenous protein complexes with restricted mobility. Potential candidates are VEGF receptors (see Fig. 3c, d)^21,22,24,68^, the PDZ domain protein GAIP-interacting protein C-terminal (GIPC)^69,70^, or mobility-restricting cellular structures such as the cytoskeleton or clathrin coated pits^52–57^. In cells coexpressing HA-ENG/myc-NRP1, addition of VEGF-A (but not BMP9) led to a further reduction in *R*_f_ of myc-NRP1 following immobilization of HA-ENG with no effect on *D* (Fig. 2d, e), suggesting that VEGF-A enhances the stable interactions between NRP1 and ENG. These findings are in accord with qualitative studies which showed that ENG interacts with NRP1^41^. Our findings demonstrate this complex formation quantitatively, show that the interactions are stable, and indicates they are enhanced by VEGF-A. Of note, BMP9, which binds to ENG, did not alter the interactions between ENG and NRP1 (Fig. 2d, e). Together with the similar observations on the lack of effect of BMP9 on ENG/VEGFR2 interactions (Fig. 3), this suggests that any effects of ENG on VEGF-A signaling are independent of BMP9 binding.

We next conducted analogous studies on the interactions between ENG and VEGFR2 at the cell surface. The patch/FRAP experiments (Fig. 3) on the effects of coexpressing HA-ENG (without and with IgG crosslinking) and/or VEGF-A or BMP9 on the lateral diffusion of myc-VEGFR2 exhibited the same pattern observed for ENG/NRP1 interactions. These studies demonstrated the formation of stable ENG/VEGFR2 complexes, deduced from the reduction in *R*_f_ of myc-VEGFR2 upon immobilization of HA-ENG with no effect on *D*. As in the case of ENG/NRP1 complexes, the interactions between ENG and VEGFR2 were enhanced by VEGF-A (but not by BMP9), in line with former reports on interactions between these two receptors^42^ and on effects of *NRP1* knockdown or overexpression on VEGFA-mediated VEGFR2 signaling^71^. Moreover, a reduction in *R*_f_ of singly-expressed myc-VEGFR2 upon incubation with VEGF-A was evident as in the case of singly-expressed myc-NRP1, suggesting ligand-mediated binding to other membrane associated proteins and/or cellular structures with restricted mobility, as explained in the section above. We have also explored the interactions between VEGFR2 and NRP1. This pair of receptors also formed complexes which were stable on the FRAP timescale and enhanced by VEGF-A, as demonstrated by the finding that IgG-mediated immobilization of HA-VEGFR2 coexpressed with myc-NRP1 markedly reduced *R*_f_ of the latter (Fig. 4a, b), with a stronger reduction in the presence of VEGF-A. These results are in line with the reports that NRP1 enhances VEGFR2 dimerization and VEGF-A binding, as well as VEGF-A-mediated activation of multiple cell signaling pathways^12–14,23^.

In view of the finding that both NRP1 and VEGFR2 form stable complexes with ENG, we hypothesized that a triple complex containing all three receptors may be formed. To test this hypothesis, we explored the effects of overexpressing untagged ENG on HA-VEGFR2/myc-NRP1 complex formation (Fig. 4c, d). As expected for co-binding of the latter tagged proteins to ENG, their coexpression with untagged ENG reduced *R*_f_ (but not *D*) of myc-NRP1, an effect that was augmented by immobilization of HA-VEGFR2 (Fig. 4c), revealing that ENG may serve as a bridge and enhance the formation of stable complexes between VEGFR2 and NRP1. Next, we employed competition patch/FRAP experiments to determine whether there is an overlap between the ENG binding sites for NRP1 and VEGFR2 (Supplementary Fig. 4). Overexpression of untagged NRP1 failed to affect the interactions between HA-ENG and myc-VEGFR2, supporting the conclusion that ENG can increase the association between NRP1 and VEGFR2 by binding them at non-overlapping sites. Regarding the NRP1 domain that interacts with ENG, as NRP1 interacts with VEGFR2 *via* its FV/FVIII domain^72^, NRP1 may interact with ENG *via* its MAM domain in the extracellular and membrane proximal regions, which has been hypothesized to mediate interactions of NRP1 with other transmembrane receptors^73,74^.

**Fig. 9:**
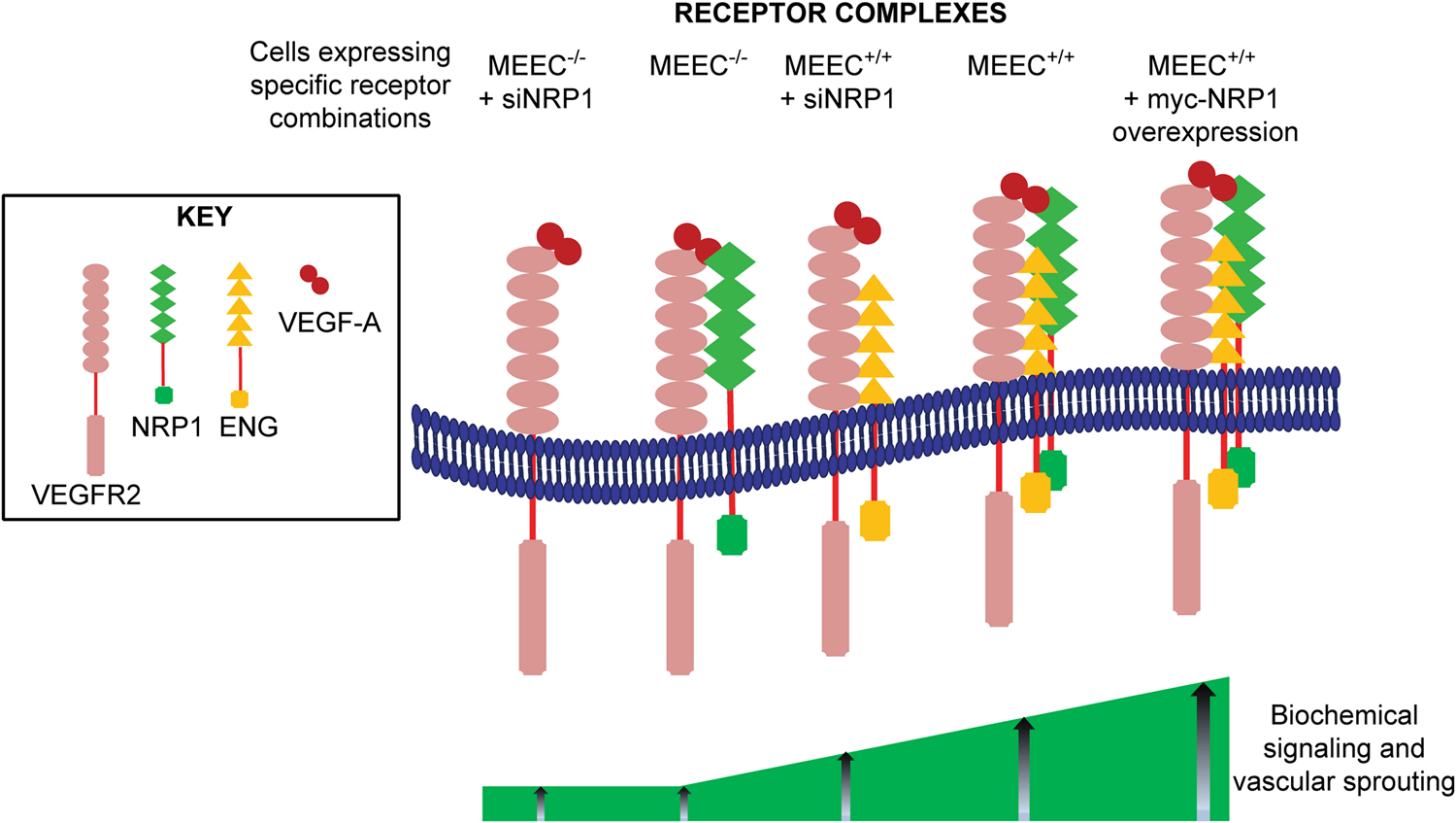
Schematic model for the interactions of ENG with VEGFR2 and NRP1 and their effects on VEGF-A signaling and sprouting. The receptors, which form homodimers, are depicted as monomers for simplicity. Complex formation of VEGFR2 with ENG is required for signaling, which bridges between VEGFR2 and NRP1 to enhance signaling and sprouting. The receptors expressed by each cell line under specific conditions are shown at the top (MEEC^-/-^ cells treated with si*NRP1* express mainly VEGFR2, MEEC^-/-^ cells express VEGFR2 and NRP1, MEEC^+/+^ cells transfected by si*NRP1* express VEGFR2 and ENG, while MEEC^+/+^ express all three receptors, with overexpression of NRP1 in MEEC^+/+^ transfected with NRP1). As shown by the two cell lines at the left, VEGFR2 alone (Figs. 6 and 8) or together with NRP1 (Figs. 5 and 7) does not induce significant signaling or sprouting upon VEGF-A binding. Expression of ENG with VEGFR2 (MEEC^+/+^ + si*NRP1*) results in a mild response to VEGF-A in both biochemical signaling (Fig. 6) and sprouting (Fig. 8). These responses are enhanced by expression of NRP1 along with ENG and VEGFR2 (MEEC^+/+^ cells; Figs. 5 and 7, or by transfection of MEEC^-/-^ cells with both ENG and NRP1 – Supplementary Fig. 7), and overexpression of NRP1 further increases the signaling and biological response (Figs. 5 and 7, and Supplementary Fig. 7). Together with our finding that ENG binds VEGFR2 and NRP1 at non-overlapping sites (Fig. 4 and Supplementary Fig. 4), we propose that the tripartite complex formed ENG bridging between VEGFR2 and NRP1 regulates the intensity of the VEGF-A-induced signaling and sprouting of ECs.

Taken together, the biophysical studies suggest that complexes between the three receptors as pairs or tripartite complexes are present to some degree prior to ligand stimulation, and are stable at least on the FRAP timescale. Moreover, they are enhanced by VEGF-A, which binds to VEGFR2 and NRP1, but not to ENG. ENG apparently binds each of the other receptors, and can serve as a scaffold to bring them together (Fig. 9).

The formation of tripartite complexes between ENG, NRP1 and VEGFR2 may regulate VEGF-A signaling and biological effects (sprouting) in ECs. To examine the modulation of VEGF-A signaling and VEGF-A-induced sprouting by complex formation between the above receptors, we conducted signaling and sprouting studies on MEEC^+/+^ and MEEC^-/-^ cells, which do or do not express ENG, respectively. To modify NRP1 expression in these two cell lines, we employed either NRP1 (or ENG) overexpression or siRNA-mediated knockdown of NRP1. ENG was required to enable VEGF-A-induced pVEGFR2 and pErk1/2 formation, both in MEEC^+/+^ and in MEEC^-/-^ cells expressing a lower level of endogenous NRP1 or overexpressing both ENG and NRP1 (Figs. 5, 6, and Supplementary Fig. 7). Accordingly, ENG was also required for VEGF-A stimulation of MEEC sprouting under similar conditions (Figs. 7, 8, and Supplementary Fig. 10). These findings indicate that in the absence of ENG, VEGFR2/NRP1 complexes (Fig. 4) do not signal significantly in response to VEGF-A.

A role for NRP1 as a modulator of VEGF-A signaling in ECs is apparent from the results of studies involving overexpression or knockdown of *NRP1*. NRP1 overexpression elevated VEGF-A-induced signaling to pVEGFR2 and pErk1/2 in MEEC^+/+^ cells, while in MEEC^-/-^ cells NRP1 induced this effect only when cotransfected with ENG (Fig. 5 and Supplementary Fig. 7). Moreover, si*NRP1* effectively inhibited VEGF-A-mediated pVEGFR2 and pErk1/2 in MEEC^+/+^ cells (Fig. 6). In sprouting assays, NRP1 overexpression (without VEGF-A) already induced a mild but significant increase of sprouting in the presence or absence of ENG (MEEC^+/+^ and MEEC^-/-^ cells) (Fig. 7), while si*NRP1* had no effect (Fig. 8). Since the level of endogenous NRP1 in MEEC^-/-^ cells is lower than in MEEC^+/+^ cells (Supplementary Fig. 5), this is suggestive of a requirement for NRP1 expression to exceed a certain threshold to induce sprouting, as demonstrated by the ability of VEGF-A to induce sprouting of MEEC^-/-^ cells cotransfected with both NRP1 and ENG (Supplementary Fig. 10). Thus, in the presence of ENG (MEEC^+/+^ cells or ENG-transfected MEEC^-/-^ cells), NRP1 overexpression potentiated VEGF-A signaling, as evident by higher levels of pVEGFR2 and pErk1/2, concomitantly enhancing sprouting (Figs. 5, 7, and Supplementary Figs. 7 and 10). The correlation between signaling to Erk1/2 and sprouting is supported by the inhibition of both pErk1/2 formation and MEEC^+/+^ sprouting by a MEK1/2 inhibitor (U0126) (Supplementary Fig. 9), in accord with earlier reports on VEGF-A-mediated signaling, sprouting and angiogenesis^9,63,64^. Further evidence for the modulation of VEGF-A signaling by NRP1 in ENG-expressing cells is provided by *NRP1* knockdown in MEEC^+/+^ cells (Figs. 6 and 8). Here, si*NRP1* reduced pVEGFR2 formation and abrogated pErk1/2 formation in response to VEGF-A (Fig. 6). In accord with these findings, *NRP1* knockdown attenuated the ability of VEGF-A to induce sprouting of MEEC^+/+^ cells (Fig. 8). These findings are in line with the reported role of Erk1/2 in EC proliferation and sprouting^61,75^. Based on these studies, we propose the model depicted in Fig. 9. In this model, the formation of specific complexes between VEGFR2, NRP1 and ENG is intertwined with VEGF-A signaling output and its biological outcome in ECs (sprouting). Thus, increased levels of signaling and sprouting are attained upon formation of distinct complexes. Four elements appear to play a role in the VEGF-A induced effects: VEGFR2, ENG, NRP1 and VEGF-A. As ENG is required for VEGF-A-mediated signaling and sprouting of the MEEC cells, VEGFR2/NRP1 complexes fall short of inducing VEGF-A-mediated signaling or sprouting, but the expression of NRP1 together with ENG results in maximal signaling, which also correlates with increased receptor complex formation. At this stage, we cannot exclude the possibility that the receptor interactions and their outcome may apply also to NRP2 and VEGFR1; however, while there is evidence that NRP2 may also be involved in EC sprouting^76^, this is not the case with VEGFR1^5,6^. Of note, cooperation between all three receptors and VEGF-A may also characterize malignant states, since NRP1, VEGFR2, ENG and VEGF-A are overexpressed in cancer;^4,77–86^ this was shown to be prominent in angiogenesis, which has a key role in tumor formation and development. Moreover, our demonstration that ENG in coordination with NRP1 and VEGFR2 is required for maximal VEGF-A signaling and sprouting has implications to HHT, which is caused primarily by loss of function mutations in TGF-β/BMP superfamily signaling components, including ENG^87,88^. Thus, loss of ENG function could alter VEGF-A signaling *via* VEGFR2 or the balance of this signaling pathway relative to other pro- and anti-angiogenic signals contributing to the pathogenesis of HHT. The current findings suggest the potential of drugs that interfere with the formation of the tripartite ENG/VEGFR2/NRP1 receptor complex and/or its association with VEGF-A.

## Methods

### Reagents

Recombinant human VEGF-A (VEGF_165_; cat. #100-20) and BMP9 (cat. #120-07) were from PeproTech (Rocky Hill, NJ). U0126, a MEK1/2 inhibitor (cat. #9903 S) was from Cell Signaling Technology (Danvers, MA). Bovine serum albumin (BSA, fraction V; cat. #10-735-094-001) was from Roche Diagnostics (Manheim, Germany). Dulbecco’s modified Eagle’s medium (DMEM; cat. #01-052-1 A) and cell culture reagents (fetal calf serum, L-glutamine, penicillin-streptomycin, sodium pyruvate) were from Biological Industries Israel (Beit Haemek, Israel-Sartorius group). MCDB-131 medium (cat. #10372019) was from Invitrogen-ThermoFisher Scientific (Waltham, MA). Hanks’ balanced salt solution (HBSS) with Ca^2+^/Mg^2+^ without phenol red (cat. #009015237500), 4-(2-hydroxyethyl)-1-piperazineethanesulfonic acid (HEPES, 1 M, pH 7.3; cat. #000773233100) and phosphate buffered saline (cat. #001623237500) were from Bio-Lab Ltd. (Jerusalem, Israel). Protease inhibitor cocktail (cat. #P8340), heparin (cat #H3149), endothelial cell growth supplement (ECGS; cat. #02-102), Na_3_VO_4_, Triton X-100 (cat. #X100) and sodium deoxycholate (cat. #D6750) were from Sigma-Aldrich (St. Louis, MO). Opti-MEM (cat. #11058021) was from Gibco Life Technologies (Carlsbad, CA), and methylcellulose (cat. #M0512) was from Sigma-Aldrich. Methocel was prepared by dissolving 1.2 g methylcellulose in 100 ml complete cell growth medium. Matrigel growth factor-reduced basement membrane (cat #354230) was from Corning (Corning, NY).

### Antibodies

Murine monoclonal anti-myc tag (αmyc, cat. #626802) 9E10 IgG^89^ and HA.11 rabbit polyclonal IgG to the HA tag (αHA, cat. #902302) were from BioLegend (San Diego, CA). 12CA5 murine monoclonal anti-influenza hemagglutinin tag (αHA) IgG (cat. #11-66-606-001) was from Roche Diagnostics. Fab’ fragments were prepared from 9E10 or 12CA5 by pepsin digestion^90^. Rabbit IgG anti-myc tag (cat. #ab9106) was from Abcam (Cambridge, UK). Alexa Fluor (Alexa) 488-goat anti rabbit (GαR) IgG (cat. #R37116), Alexa 546-goat anti mouse (GαM) F(ab’)_2_ (cat. #A-11018) and Alexa 488-GαR F(ab’)_2_ (cat. #A-11070) were from Invitrogen-Molecular Probes (Eugene, OR). Fluorescent F(ab′)_2_ were converted to monovalent Fab′ by reduction with 2-mercaptoethanol followed by alkylation with iodoacetamide^91^. Normal goat γ-globulin (NGG; cat. #005-000-002), peroxidase-conjugated GαM (cat. #115-035-062) and GαR (cat. #111-035-144) IgGs were from Jackson ImmunoResearch Laboratories (West Grove, PA). Rabbit antibodies to phospho (p) VEGFR2 (Tyr1175; cat. #2478), total (t) VEGFR2 (cat. #2479), and total Erk1/2 (cat. #9102) were from Cell Signaling Technology. Murine monoclonal antibody to pErk1/2 (cat. #M8159) was from Sigma-Aldrich. Rabbit monoclonal antibody to total (t) NRP1 (cat. #ab81321) was from Abcam, and murine anti-β-actin (cat. #08691001) from MP Biomedicals (Solon, OH).

### Plasmids and small interfering RNA (siRNA)

Expression vectors encoding N-terminally HA- or myc-tagged human VEGFR2 in pCMV3 (cat. #HG10012-NY and HG10012-NM, respectively) were purchased from Sino Biologicals (Beijing, China). Their activity was validated as described in Supplementary Fig. 1. HA-tagged endoglin-L (ENG) and untagged ENG in pDisplay vector, described earlier^50,92^, were donated by Prof. G. Blobe (Duke University, Durham, NC). N-terminally HA-tagged human ACVR2B (with the HA tag inserted by overlapping PCR after nucleotide 66) in pCDNA3.1 was donated by Prof. P. Knaus (Free University of Berlin, Germany). Human NRP1 in pcDNA3.1 Hygro vector^93^ was donated by Prof. Gera Neufeld (Technion, Haifa, Israel). N-terminal myc tag was introduced by a three-step PCR procedure: (1) The T7 promoter primer was used as the forward primer, with a reverse primer recognizing part of the myc tag sequence (underlined) (5’-GGCGCTTTCGCGAACAAAAACTC-3’). (2) A second PCR reaction employed a forward primer recognizing the rest of the myc tag (underlined; 5’-ATCTCAGAAGAGGATCTGAACGATAAATGTGGC-3’) and a reverse primer recognizing the region encoding the NRP1 C-terminus (5’-TATTCGGAGGCATGACTCGAGGGG-3’). (3) The PCR products of steps 1 and 2 served as a template, using 5’-GAACAAAAACTCATCTCAGAAGAGGATCTG-3’ as a forward primer and its complementary strand as a reverse primer, to generate one product containing NRP1 with the myc tag sequence inserted after nucleotide 23. The final PCR product was digested with BamHI and XhoI and inserted into similarly digested pcDNA3.1 Hygro. All constructs were verified by sequencing. ON‐TARGETplus SMARTpool murine siRNA to *NRP1* (cat. #L-002000-00-0020) and non-targeting pool (siScrambled; cat. #D-001810-10-05) siRNA were purchased from Dharmacon (Lafayette, CO).

### Cell culture and transfections

COS7 cells (ATCC, American Type Culture Collection, cat. #CRL-1651) and HEK293T cells (ATCC, cat. #CRL-3216) were grown in DMEM supplemented with 10% FCS, penicillin, streptomycin and 2 mM L-glutamine. Murine embryonic endothelial cells (MEEC) from WT ENG (MEEC^+/+^) and ENG-null (MEEC^-/-^) mice^48^ were a gift from E. Dejana, Milan, Italy; they were grown in MCDB-131 medium supplemented with 10% FCS, 2 mM L-glutamine, 1 mM sodium pyruvate, 100 μg/ml heparin, and 50 μg/ml ECGS. The MEEC lines were cultured in flasks coated with Gibco attachment factor 1X (cat. #S-006-100). All cells were grown at 37 °C with 5% CO_2_. The HEK293T human cell line was authenticated by STR analysis at the Genomics Center of the Biomedical Core Facility, Technion, Haifa, Israel. All cells were routinely analyzed by RT-PCR for mycoplasma contamination and found to be clean.

For Patch/FRAP experiments, COS7 cells grown on glass coverslips in 6-wells plates were transfected by TransIT-LT1 transfection reagent (cat. #MIR 2304; Mirus Bio LLC, Madison, WI) with different combinations of vectors encoding myc- and HA-tagged (or untagged) receptor constructs. The amounts of the various vectors in the transfection were adjusted to yield similar cell-surface expression levels, determined by quantitative immunofluorescence using the FRAP setup to measure the fluorescence intensity at the cell surface^47^, employing 200 ng plasmid DNA for ENG (untagged or HA-tagged), 150 ng for NRP1 (untagged or myc-tagged), and 1 μg VEGFR2 (myc- or HA-tagged). The total DNA level was complemented by empty vector to 2 μg.

For signaling assays, MEECs were grown in attachment factor-coated 6-well plates and transfected (24 or 48 h) with 1 μg/dish myc-NRP1 expression vector or empty vector using Lipofectamine 3000 (cat. #L3000001; Invitrogen-ThermoFisher Scientific) according to the manufacturer’s instructions. Transfection for biological assays was similar, except that the cells were plated on 60 mm dishes and the amount of DNA was adjusted accordingly. For studies on the effects of silencing *NRP1*, MEECs grown in 6-well plates were transfected by Lipofectamine 3000 with 50 nM si*NRP1* or siScrambled, and the amount of the siRNA was adjusted for cells grown on 60 mm plates for biological studies. For all experiments, cells were assayed 24-48 h post-transfection, as mentioned in the figure legends.

### Antibody labeling of cell-surface epitope tagged receptors and IgG-mediated crosslinking

At 24 h post-transfection, COS7 cells plated on glass coverslips were transfected with various combinations of expression vectors encoding the above myc- and/or HA-tagged receptors were serum-starved (30 min, 37 °C), washed with cold HBSS supplemented with 20 mM HEPES (pH 7.2) and 2% BSA (HBSS/HEPES/BSA), and blocked by incubation with NGG (200 μg/ml, 30 min, 4 °C). For FRAP studies on singly-expressed receptors, they were labeled consecutively at 4 °C (to enable exclusive cell surface labeling) in HBSS/HEPES/BSA (45 min incubations) with: (i) monovalent murine Fab’ αmyc or Fab’ of 12CA5 αHA (40 μg/ml); (ii) Alexa 546-Fab’ GαM (40 μg/ml). For studies on the interactions between coexpressed receptors by patch/FRAP, labeling was with: (i) monovalent mouse Fab’ αmyc (40 μg/ml) together with HA.11 rabbit IgG αHA (20 μg/ml) and (ii) Alexa Fluor 546-Fab’ GαM (40 μg/ml) together with Alexa Fluor 488-IgG GαR (20 μg/ml). This protocol leads to IgG crosslinking and immobilization of the HA-tagged receptors, while the myc-tagged receptors, whose lateral diffusion is then measured by FRAP, are labeled exclusively by monovalent Fab’ fragments. To study the effects of ligands, VEGF-A (50 ng/ml) or BMP9 (5 ng/ml) were added at the NGG blocking step, and maintained during all following labeling steps and FRAP measurements.

### FRAP and Patch/FRAP measurements

Coexpressed epitope-tagged receptors labeled by fluorescent IgG and Fab’ were subjected to FRAP and patch/FRAP studies^43,47,59^. The studies were conducted at 15 °C, replacing samples within 20 min, to minimize internalization. An argon-ion laser beam (Innova 70 C, Coherent, Santa Clara, CA) was focused through a fluorescence microscope (Axioimager.D1, Carl Zeiss MicroImaging, Jena, Germany) to a Gaussian spot of 0.77 ± 0.03 μm (plan-apochromat 63x/1.4 NA oil-immersion objective). After a brief measurement at monitoring laser intensity (528.7 nm, 1 μW), a 5 mW pulse (20 ms) bleached 60–75% of the fluorescence in the illuminated region, and fluorescence recovery was followed at the monitoring intensity. The *D* and *R*_f_ values were extracted from the FRAP curves by nonlinear regression analysis, fitting to a lateral diffusion process^94^. Patch/FRAP studies were performed similarly, except that the FRAP measurements of the Fab’-labeled receptors were conducted on cells where the HA-tagged receptors were immobilized by IgG crosslinking^43,47,59^.

### Signaling assays and Western blotting

At 24-48 h post-transfection, MEEC^+/+^ or MEEC^-/-^ grown overnight in attachment factor-coated 6-well plates were serum starved (30 min, 1% FCS) and incubated (5 min) with or without VEGF-A (50 ng/ml). In experiments with the MEK1/2 inhibitor U0126, it was added at the start of starvation (10 μM, diluted 1:1000 from a stock solution in dimethylsulfoxide (DMSO)). The cells were lysed on ice (30 min) with RIPA lysis buffer (137 mM NaCl, 20 mM Tris-HCl, 2 mM EDTA, 0.5% SDS, 7 mM sodium deoxycholate, 1% TritonX-100, 10% glycerol, 1% protease inhibitor cocktail and 0.1 mM Na_3_VO_4_). The lysates were subjected to low speed centrifugation, followed by SDS-PAGE (7.5% polyacrylamide) and immunoblotting^42^. The blots were incubated overnight (4 °C) with primary antibodies to rabbit anti-pVEGFR2 (1:1000), rabbit anti-tVEGFR2 (1:1000), mouse anti-pErk1/2 (1:10000), rabbit anti tErk1/2 (1:1000), rabbit anti-tNRP1 (1:1000), mouse αmyc (1:1000), rabbit HA.11 IgG (1:1000) or mouse anti-β-actin (1:50000), followed by peroxidase GαR or GαM IgG (1:5000, 1 h, 22 °C). The bands were visualized by ECL with Clarity ECL substrate (cat. #1705060, Bio-Rad, Hercules, CA), recorded using ChemiDoc Touch imaging system (Bio-Rad) and quantified by Image Lab (Bio-Rad).

### Spheroid sprouting assay

Sprouting of MEECs was measured using a modified protocol previously reported^95,96^. Briefly, MEEC^+/+^ or MEEC^-/-^ were cultured 24 h in 60 mm dishes to reach 70−80% confluence. For studies on NRP1 overexpression, the cells were then transfected with myc-NRP1 or empty vector; for *NRP1* silencing, they were transfected with si*NRP1* (replaced by siScrambled for control) as described under cell culture and transfection. At 24 h post-transfection, cells were trypsinized, lifted in full medium, counted and cultured in hanging drops of 25 μl medium (80% full medium and 20% Methocel) containing 2 × 10^5^ cells in Petri dishes. After 24 h, the spheroids were collected, plated on 96-well plates coated with 150 μl growth factor-reduced Matrigel (50 μL per well), and incubated 1 h at 37 °C. VEGF-A (100 ng/ml) was added (in some cases, along with 10 μM U0126) where indicated, and the spheroids were allowed to sprout on Matrigel for 24 h. Phase contrast images of the sprouting spheroids were taken with Olympus IX81 microscope using 10× objective (Olympus Corporation, Tokyo, Japan). The area of sprouts per spheroid was quantified using Image Pro Plus software (Media Cybernetics, Silver Spring, MD, U.S.A.). The acquired images were processed and optimized using tophat and enhancement filters to outline the sprouts area. The sprouts area per spheroid was then measured using the count/size function, followed by subtraction of the center body of the spheroid.

### RT-qPCR assay

MEEC^+/+^ or MEEC^-/-^ cells grown in attachment factor-coated 60 mm plates were subjected to total RNA isolation using EZ-RNA kit (cat. #20-400-100, Biological Industries Israel) according to the manufacturer’s instructions. RNA was reverse transcribed to cDNA using Verso cDNA Synthesis Kit (cat. #AB-1453-B, Thermo Fisher Scientific). The mRNA levels of endogenous *ENG, VEGFR2 (KDR) and NRP1* were determined in triplicate by RT-qPCR using KAPA SYBR FAST ABI Prism qPCR kit (cat. #KK-KK4604, Kapa Biosystems-Roche, Wilmington, MA), and quantified with Applied Biosystems 7300 Real-Time PCR System Software (Thermo Fisher Scientific). Relative mRNA expression values were calculated based on the comparative threshold cycle (C_T_) method^97^, normalizing the data to mouse GAPDH. The sequences of the primers used for each receptor are listed in Table 1.

**Table 1:** Sequences of the primer pairs used for RT-qPCR of the murine receptor genes.

Gene	Forward primer (5’ to 3’)	Reverse primer (5’ to 3’)
*ENG*	AGGGGTGAGGTGACGTTTAC	GTGCCATTTTGCTTGGATGC
*NRP1*	GGCTCTGAAGACCTGGCAAT	GTTCATCCTGGACAGTGGCA
*VEGFR2*	TTCACAGTCGGGTTACAGGC	TCTCACAATTCTTCGGCCCC
*GAPDH*	TTCACCACCATGGAGAAGGC	AGTGATGGCATGGACTGTGG

### Statistics and reproducibility

All the numbers of independent measurements are given in the figure legends. FRAP data are representative of at least 27 experiments in each case, conducted on different cells. Western blotting experiments are from at least four independent experiments, and sprouting studies are from at least 11 spheroids per condition, derived from three independent experiments. Statistical analysis was done by Prism9 (GraphPad Software, San Diego, CA). Significant differences between multiple data sets were evaluated by one-way ANOVA followed by post hoc Bonferroni test. Student’s *t* test was used to calculate the significance of the difference between two groups. Data are presented throughout as mean ± SEM. *p* values below 0.05 were defined as statistically significant. All attempts at replication were successful, with similar results.

### Reporting summary

Further information on research design is available in the Nature Portfolio Reporting Summary linked to this article.

### Supplementary information

Supplementary Information

Description of Additional Supplementary Files

Supplementary Data

Reporting Summary

## Data Availability

All data generated or analyzed during this study are included in this article and its supplementary information files. The Supplementary Information file contains all supplementary figures (Supplementary Figs. 1-10) and the original uncropped Western blots (Supplementary Fig. 11). The source data behind all graphs in the manuscript are in the Supplementary Data file. All other data are available from the corresponding author on reasonable request.

## References

[1] Carmeliet P, Jain RK (2011). Molecular mechanisms and clinical applications of angiogenesis. Nature.

[2] Welti J, Loges S, Dimmeler S, Carmeliet P (2013). Recent molecular discoveries in angiogenesis and antiangiogenic therapies in cancer. J. Clin. Investig..

[3] Tian H (2017). Endoglin mediates vascular maturation by promoting vascular smooth muscle cell migration and spreading. Arterioscler. Thromb. Vasc. Biol..

[4] Nagy JA, Dvorak AM, Dvorak HF (2007). VEGF-A and the induction of pathological angiogenesis. Annu. Rev. Pathol..

[5] Gerhardt H (2003). VEGF guides angiogenic sprouting utilizing endothelial tip cell filopodia. J. Cell Biol..

[6] Simons M, Gordon E, Claesson-Welsh L (2016). Mechanisms and regulation of endothelial VEGF receptor signalling. Nat. Rev. Mol. Cell Biol..

[7] Patel SA (2023). Molecular mechanisms and future implications of VEGF/VEGFR in cancer therapy. Clin. Cancer Res..

[8] Shibuya M (2013). Vascular endothelial growth factor and its receptor system: physiological functions in angiogenesis and pathological roles in various diseases. J. Biochem..

[9] Wang X, Bove AM, Simone G, Ma B (2020). Molecular bases of VEGFR-2-mediated physiological function and pathological role. Front. Cell Dev. Biol..

[10] Brozzo MS (2012). Thermodynamic and structural description of allosterically regulated VEGFR-2 dimerization. Blood.

[11] Ruch C, Skiniotis G, Steinmetz MO, Walz T, Ballmer-Hofer K (2007). Structure of a VEGF-VEGF receptor complex determined by electron microscopy. Nat. Struct. Mol. Biol..

[12] Zachary I (2003). VEGF signalling: integration and multi-tasking in endothelial cell biology. Biochem. Soc. Trans..

[13] Huang K, Andersson C, Roomans GM, Ito N, Claesson-Welsh L (2001). Signaling properties of VEGF receptor-1 and -2 homo- and heterodimers. Int. J. Biochem. Cell Biol..

[14] Abhinand CS, Raju R, Soumya SJ, Arya PS, Sudhakaran PR (2016). VEGF-A/VEGFR2 signaling network in endothelial cells relevant to angiogenesis. J. Cell Commun. Signal..

[15] Parker MW, Xu P, Li X, Vander Kooi CW (2012). Structural basis for selective vascular endothelial growth factor-A (VEGF-A) binding to neuropilin-1. J. Biol. Chem..

[16] Peach CJ (2018). Molecular pharmacology of VEGF-A isoforms: Binding and signalling at VEGFR2. Int. J. Mol. Sci..

[17] Holmes K, Roberts OL, Thomas AM, Cross MJ (2007). Vascular endothelial growth factor receptor-2: structure, function, intracellular signalling and therapeutic inhibition. Cell. Signal..

[18] Koch S, Tugues S, Li X, Gualandi L, Claesson-Welsh L (2011). Signal transduction by vascular endothelial growth factor receptors. Biochem. J..

[19] Fujisawa H (1997). Roles of a neuronal cell-surface molecule, neuropilin, in nerve fiber fasciculation and guidance. Cell Tissue Res..

[20] Chen H, He Z, Bagri A, Tessier-Lavigne M (1998). Semaphorin-neuropilin interactions underlying sympathetic axon responses to class III semaphorins. Neuron.

[21] King C, Wirth D, Workman S, Hristova K (2018). Interactions between NRP1 and VEGFR2 molecules in the plasma membrane. Biochim. Biophys. Acta Biomembr..

[22] Soker S, Fidder H, Neufeld G, Klagsbrun M (1996). Characterization of novel vascular endothelial growth factor (VEGF) receptors on tumor cells that bind VEGF165 via its exon 7-encoded domain. J. Biol. Chem..

[23] Soker S, Takashima S, Miao HQ, Neufeld G, Klagsbrun M (1998). Neuropilin-1 is expressed by endothelial and tumor cells as an isoform-specific receptor for vascular endothelial growth factor. Cell.

[24] Sarabipour S, Mac Gabhann F (2018). VEGF-A121a binding to Neuropilins - A concept revisited. Cell Adh. Migr..

[25] Vander Kooi CW (2007). Structural basis for ligand and heparin binding to neuropilin B domains. Proc. Natl. Acad. Sci. USA..

[26] Wang L, Zeng H, Wang P, Soker S, Mukhopadhyay D (2003). Neuropilin-1-mediated vascular permeability factor/vascular endothelial growth factor-dependent endothelial cell migration. J. Biol. Chem..

[27] Kawamura H (2008). Neuropilin-1 in regulation of VEGF-induced activation of p38MAPK and endothelial cell organization. Blood.

[28] Valluru M, Staton CA, Reed MW, Brown NJ (2011). Transforming growth factor-beta and endoglin signaling orchestrate wound healing. Front. Physiol..

[29] Gougos A, Letarte M (1988). Biochemical characterization of the 44G4 antigen from the HOON pre-B leukemic cell line. J. Immunol..

[30] Pomeraniec L, Hector-Greene M, Ehrlich M, Blobe GC, Henis YI (2015). Regulation of TGF-b receptor hetero-oligomerization and signaling by endoglin. Mol. Biol. Cell.

[31] McAllister KA (1994). Endoglin, a TGF-b binding protein of endothelial cells, is the gene for hereditary haemorrhagic telangiectasia type 1. Nat. Genet..

[32] Bourdeau A, Dumont DJ, Letarte M (1999). A murine model of hereditary hemorrhagic telangiectasia. J. Clin. Investig..

[33] ten Dijke P, Goumans MJ, Pardali E (2008). Endoglin in angiogenesis and vascular diseases. Angiogenesis.

[34] Miller DW (1999). Elevated expression of endoglin, a component of the TGF-b-receptor complex, correlates with proliferation of tumor endothelial cells. Int. J. Cancer.

[35] Fonsatti E, Altomonte M, Nicotra MR, Natali PG, Maio M (2003). Endoglin (CD105): a powerful therapeutic target on tumor-associated angiogenetic blood vessels. Oncogene.

[36] Ollauri-Ibanez C, Ayuso-Inigo B, Pericacho M (2021). Hot and cold tumors: is endoglin (CD105) a potential target for vessel normalization?. Cancers.

[37] Hendriksen EM (2009). Angiogenesis, hypoxia and VEGF expression during tumour growth in a human xenograft tumour model. Microvasc. Res..

[38] Li C (2003). CD105 prevents apoptosis in hypoxic endothelial cells. J. Cell Sci..

[39] Horst B (2022). Hypoxia-induced inhibin promotes tumor growth and vascular permeability in ovarian cancers. Commun. Biol..

[40] Nogues A (2020). Endoglin (CD105) and VEGF as potential angiogenic and dissemination markers for colorectal cancer. World J. Surg. Oncol..

[41] Kilari S (2022). Neuropilin-1 deficiency in vascular smooth muscle cells is associated with hereditary hemorrhagic telangiectasia arteriovenous malformations. JCI Insight.

[42] Tian H (2018). Endoglin interacts with VEGFR2 to promote angiogenesis. FASEB J..

[43] Rechtman MM, Nakaryakov A, Shapira KE, Ehrlich M, Henis YI (2009). Different domains regulate homomeric and heteromeric complex formation among type I and type II transforming growth factor-b receptors. J. Biol. Chem..

[44] Ehrlich M, Gutman O, Knaus P, Henis YI (2012). Oligomeric interactions of TGF-b and BMP receptors. FEBS Lett..

[45] Marom B, Heining E, Knaus P, Henis YI (2011). Formation of stable homomeric and transient heteromeric bone morphogenetic protein (BMP) receptor complexes regulates Smad protein signaling. J. Biol. Chem..

[46] Tazat K, Hector-Greene M, Blobe GC, Henis YI (2015). TbRIII independently binds type I and type II TGF-b receptors to inhibit TGF-b signaling. Mol. Biol. Cell.

[47] Szilagyi SS, Amsalem-Zafran AR, Shapira KE, Ehrlich M, Henis YI (2022). Competition between type I activin and BMP receptors for binding to ACVR2A regulates signaling to distinct Smad pathways. BMC Biol..

[48] Pece-Barbara N (2005). Endoglin null endothelial cells proliferate faster and are more responsive to transforming growth factor b1 with higher affinity receptors and an activated Alk1 pathway. J. Biol. Chem..

[49] Tazat K (2021). ALK1 Regulates the Internalization of Endoglin and the type III TGF-b Receptor. Mol. Biol. Cell.

[50] Lee NY, Ray B, How T, Blobe GC (2008). Endoglin promotes transforming growth factor b-mediated Smad 1/5/8 signaling and inhibits endothelial cell migration through its association with GIPC. J. Biol. Chem..

[51] Yoshida A (2015). VEGF-A/NRP1 stimulates GIPC1 and Syx complex formation to promote RhoA activation and proliferation in skin cancer cells. Biol. Open.

[52] Sheetz MP, Schindler M, Koppel DE (1980). Lateral mobility of integral membrane proteins is increased in spherocytic erythrocytes. Nature.

[53] Jacobson K, Ishihara A, Inman R (1987). Lateral diffusion of proteins in membranes. Annu. Rev. Physiol..

[54] Sako Y, Kusumi A (1995). Barriers for lateral diffusion of transferrin receptor in the plasma membrane as characterized by receptor dragging by laser tweezers: fence versus tether. J. Cell Biol..

[55] Fire E, Gutman O, Roth MG, Henis YI (1995). Dynamic or stable interactions of influenza hemagglutinin mutants with coated pits. Dependence on the internalization signal but not on aggregation. J. Biol. Chem..

[56] Yao D, Ehrlich M, Henis YI, Leof EB (2002). Transforming growth factor-b receptors interact with AP2 by direct binding to b2 subunit. Mol. Biol. Cell.

[57] Freeman SA (2018). Transmembrane pickets connect cyto- and pericellular skeletons forming barriers to receptor engagement. Cell.

[58] Henis YI, Katzir Z, Shia MA, Lodish HF (1990). Oligomeric structure of the human asialoglycoprotein receptor: nature and stoichiometry of mutual complexes containing H1 and H2 polypeptides assessed by fluorescence photobleaching recovery. J. Cell Biol..

[59] Szilagyi SS, Gutman O, Henis YI (2022). Complex formation among TGF-b receptors in live cell membranes measured by patch-FRAP.. Methods Mol. Biol..

[60] Prahst C (2008). Neuropilin-1-VEGFR-2 complexing requires the PDZ-binding domain of neuropilin-1. J. Biol. Chem..

[61] Dellinger MT, Brekken RA (2011). Phosphorylation of Akt and ERK1/2 is required for VEGF-A/VEGFR2-induced proliferation and migration of lymphatic endothelium. PLoS ONE.

[62] Tian H, Mythreye K, Golzio C, Katsanis N, Blobe GC (2012). Endoglin mediates fibronectin/a5b1 integrin and TGF-b pathway crosstalk in endothelial cells. EMBO J..

[63] Wu MH, Yuan SY, Granger HJ (2005). The protein kinase MEK1/2 mediate vascular endothelial growth factor- and histamine-induced hyperpermeability in porcine coronary venules. J. Physiol..

[64] Claesson-Welsh L, Welsh MVEGFA (2013). and tumour angiogenesis. J. Intern. Med..

[65] Fire E, Zwart DE, Roth MG, Henis YI (1991). Evidence from lateral mobility studies for dynamic interactions of a mutant influenza hemagglutinin with coated pits. J. Cell Biol..

[66] Eisenberg S (2011). Raft protein clustering alters N-Ras membrane interactions and activation pattern. Mol. Cell. Biol..

[67] Saffman PG, Delbruck M (1975). Brownian motion in biological membranes. Proc. Natl. Acad. Sci. USA..

[68] Guo HF, Vander Kooi CW (2015). Neuropilin functions as an essential cell surface receptor. J. Biol. Chem..

[69] Cai H, Reed RR (1999). Cloning and characterization of neuropilin-1-interacting protein: a PSD-95/Dlg/ZO-1 domain-containing protein that interacts with the cytoplasmic domain of neuropilin-1. J. Neurosci..

[70] Wang L, Mukhopadhyay D, Xu X (2006). C terminus of RGS-GAIP-interacting protein conveys neuropilin-1-mediated signaling during angiogenesis. FASEB J..

[71] Jin Y (2017). Endoglin prevents vascular malformation by regulating flow-induced cell migration and specification through VEGFR2 signalling. Nat. Cell Biol..

[72] Gelfand MV (2014). Neuropilin-1 functions as a VEGFR2 co-receptor to guide developmental angiogenesis independent of ligand binding. eLife.

[73] Nakamura F, Tanaka M, Takahashi T, Kalb RG, Strittmatter SM (1998). Neuropilin-1 extracellular domains mediate semaphorin D/III-induced growth cone collapse. Neuron.

[74] Yelland T, Djordjevic S (2016). Crystal Structure of the Neuropilin-1 MAM Domain: completing the Neuropilin-1 *Ectodomain P*icture. Structure.

[75] Walker AMN (2021). Endothelial insulin receptors promote VEGF-A signaling via ERK1/2 and sprouting angiogenesis. Endocrinology.

[76] Dallinga MG (2021). The role of heparan sulfate and neuropilin 2 in VEGFA signaling in human endothelial tip cells and non-tip cells during angiogenesis in vitro. Cells.

[77] Jubb AM (2012). Neuropilin-1 expression in cancer and development. J. Pathol..

[78] Kim M (2017). VEGFA links self-renewal and metastasis by inducing Sox2 to repress miR-452, driving Slug. Oncogene.

[79] Modi SJ, Kulkarni VM (2019). Vascular endothelial growth factor receptor (VEGFR-2)/KDR inhibitors: Medicinal chemistry perspective. Med. Drug Discov..

[80] Minhajat R (2006). Organ-specific endoglin (CD105) expression in the angiogenesis of human cancers. Pathol. Int..

[81] Pawlak JB, Blobe GC (2022). TGF-b superfamily co-receptors in cancer. Dev. Dyn..

[82] Ollauri-Ibanez C (2020). Continuous endoglin (CD105) overexpression disrupts angiogenesis and facilitates tumor cell metastasis. Angiogenesis.

[83] Apte RS, Chen DS, Ferrara N (2019). VEGF in signaling and disease: beyond discovery and development. Cell.

[84] Ferrara N (2010). Vascular endothelial growth factor and age-related macular degeneration: from basic science to therapy. Nat. Med..

[85] Kawakami T (2002). Neuropilin 1 and neuropilin 2 co-expression is significantly correlated with increased vascularity and poor prognosis in nonsmall cell lung carcinoma. Cancer.

[86] Ferrara N, Mass RD, Campa C, Kim R (2007). Targeting VEGF-A to treat cancer and age-related macular degeneration. Annu. Rev. Med..

[87] Cunha SI, Magnusson PU, Dejana E, Lampugnani MG (2017). Deregulated TGF-b/BMP signaling in vascular malformations. Circ. Res..

[88] McDonald J, Bayrak-Toydemir P, Pyeritz RE (2011). Hereditary hemorrhagic telangiectasia: an overview of diagnosis, management, and pathogenesis. Genet. Med..

[89] Evan GI, Lewis GK, Ramsay G, Bishop JM (1985). Isolation of monoclonal antibodies specific for human c-myc proto-oncogene product. Mol. Cell. Biol..

[90] Henis YI, Moustakas A, Lin HY, Lodish HF (1994). The types II and III transforming growth factor-b receptors form homo-oligomers. J. Cell Biol..

[91] Gilboa L, Wells RG, Lodish HF, Henis YI (1998). Oligomeric structure of type I and type II TGF-b receptors: homo-dimers form in the ER and persist at the plasma membrane. J. Cell Biol..

[92] Lee NY, Blobe GC (2007). The interaction of endoglin with b-arrestin2 regulates transforming growth factor-b-mediated ERK activation and migration in endothelial cells. J. Biol. Chem..

[93] Gluzman-Poltorak Z, Cohen T, Herzog Y, Neufeld G (2000). Neuropilin-2 is a receptor for the vascular endothelial growth factor (VEGF) forms VEGF-145 and VEGF-165. J. Biol. Chem..

[94] Petersen, N. O., Felder, S. & Elson, E. L. Measurement of lateral diffusion by fluorescence photobleaching recovery. In *Handbook of Experimental Immunology* (eds D. M. Weir, L. A. Herzenberg, C. C. Blackwell, & L. A. Herzenberg) 24.21-24.23 (Blackwell Scientific Publications), (1986).

[95] Heiss M (2015). Endothelial cell spheroids as a versatile tool to study angiogenesis in vitro. FASEB J..

[96] Kannan P, Schain M, Lane DP (2022). An automated quantification tool for angiogenic sprouting from endothelial spheroids. Front. Pharmacol..

[97] Livak KJ, Schmittgen TD (2001). Analysis of relative gene expression data using real-time quantitative PCR and the 2^-DDCT^ Method. Methods.

