# Supplementary material for: NRP1 interacts with endoglin and VEGFR2 to modulate VEGF signaling and endothelial cell sprouting
Source: Commun Biol. 2024 Jan 19;7:112. doi: 10.1038/s42003-024-05798-2 (PMC10799020; doi:10.1038/s42003-024-05798-2)

SUPPLEMENTARY INFORMATION

Supplementary Figures 1 – 11, including original uncropped Western Blots

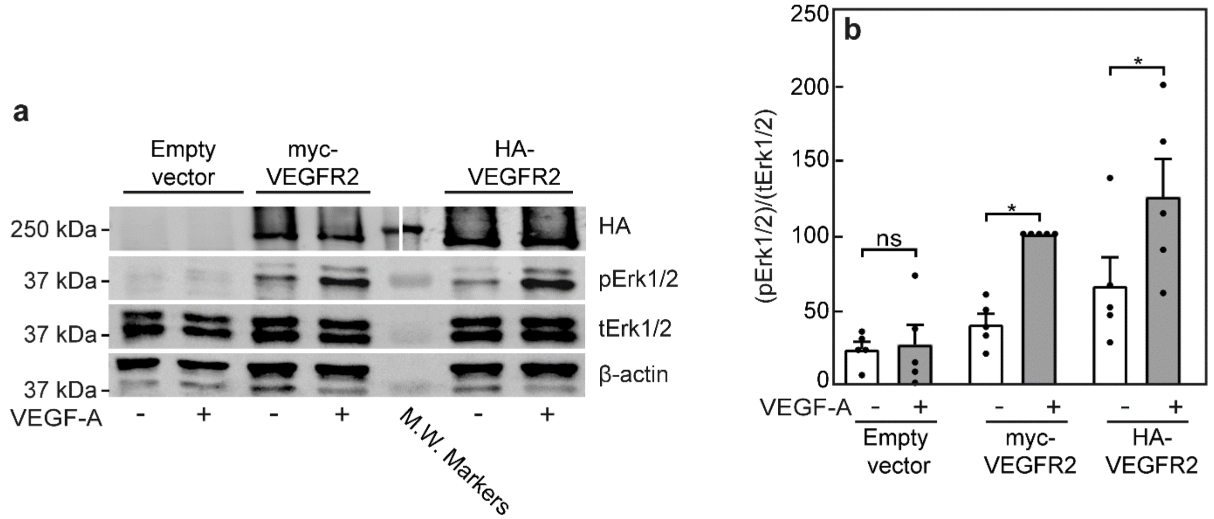

Supplementary Fig. 1 myc-VEGFR2 and HA-VEGFR2 are active in signaling. HEK293T cells were grown as described [98] in 6-well plates, transfected with 1 μg/well empty vector (control), myc-VEGFR2 or HA-VEGFR2 expression vectors using Lipofectamine 2000 (cat. #11668019, ThermoFischer Scientific). At 24 h post-transfection, the cells were stimulated (or not) with VEGF-A (50 ng/ml, 5 min), lysed, and subjected to immunoblotting using anti-myc, anti-HA, pErk1/2 or tErk1/2 antibodies. Blotting for β-actin served as loading control. **a** A representative immunoblot. **b** Quantification of VEGF-A signaling to pErk1/2. Transfection with either myc- or HA-tagged VEGFR2 enabled VEGF-A-mediated pErk1/2 formation. The bands were visualized by ECL and quantified by densitometry. Data are mean ± SEM of 5 independent experiments. The values obtained for cells transfected with myc-VEGFR2 and stimulated by VEGF-A were normalized to 100%. Asterisks indicate significant differences between the pairs indicated by the brackets (one-way ANOVA with Bonferroni post-hoc test). *, $p < 0.05$. ns = not significant.

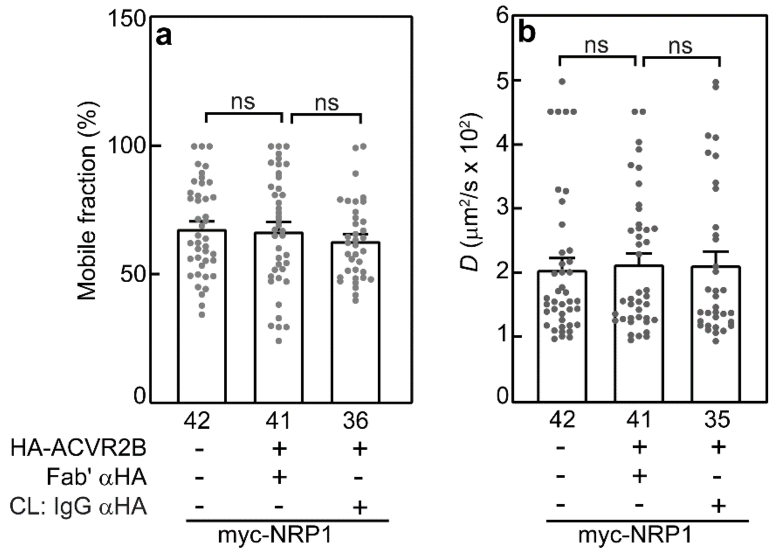

Supplementary Fig. 2 The lateral diffusion of myc-NRP1 is not affected by coexpression and IgG crosslinking of HA-ACVR2B, an unrelated HA-tagged receptor. COS7 cells were transfected with myc-NRP1 alone or together with HA-ACVR2B. After 24 h, the cells were subjected to IgG-crosslinking (CL) of HA-ACVR2B and Fab’ labeling of myc-NRP1 as described in Fig. 2. In control experiments without CL, the IgG αHA was replaced by Fab' αHA. FRAP studies to measure the lateral diffusion of myc-NRP1 were conducted as in Fig. 1. **a** Average R_f values. **b** Average D values. Bars, mean ± SEM; the number of measurements (each conducted on a different cell) is shown underneath each bar. No significant differences were detected between the R_f or D values (one-way ANOVA and Bonferroni post-hoc test; ns = not significant), demonstrating that coexpression with HA-ACVR2B, without or with IgG CL, does not affect R_f or D of myc-NRP1.

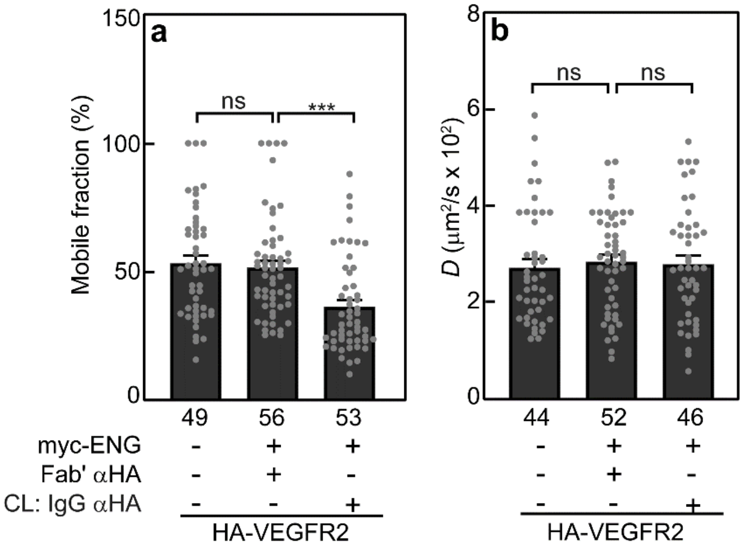

Supplementary Fig. 3 The mobile fraction of HA-VEGFR2 is reduced by IgG crosslinking of myc-ENG. Patch/FRAP studies were conducted on COS7 cells expressing HA-VEGFR2 alone or together with myc-ENG. At 24 h post-transfection, the cells were subjected to the IgG crosslinking (CL) protocol (Materials and Methods) as in Fig. 2, except that the IgG crosslinking and Fab'-labeled tags were switched: myc-ENG was patched where indicated by rabbit αmyc IgG followed by Alexa Fluor 488-IgG GαR (designated CL: IgG αmyc), and HA-VEGFR2 was labeled exclusively by monovalent murine Fab' of 12CA5 αHA followed by Alexa 546-Fab' GαM. FRAP studies were conducted as in Fig. 1, measuring the lateral diffusion of the Fab'-labeled HA-VEGFR2. **a** Average R_f values; **b** Average D values. Bars, mean ± SEM. The number of measurements (each conducted on a different cell) is shown underneath each bar. Asterisks indicate significant differences between the R_f values of the pairs indicated by brackets (***, $p < 10^{-3}$; one-way ANOVA and Bonferroni post-hoc test. ns = not significant). A similar analysis of the D values showed no significant differences.

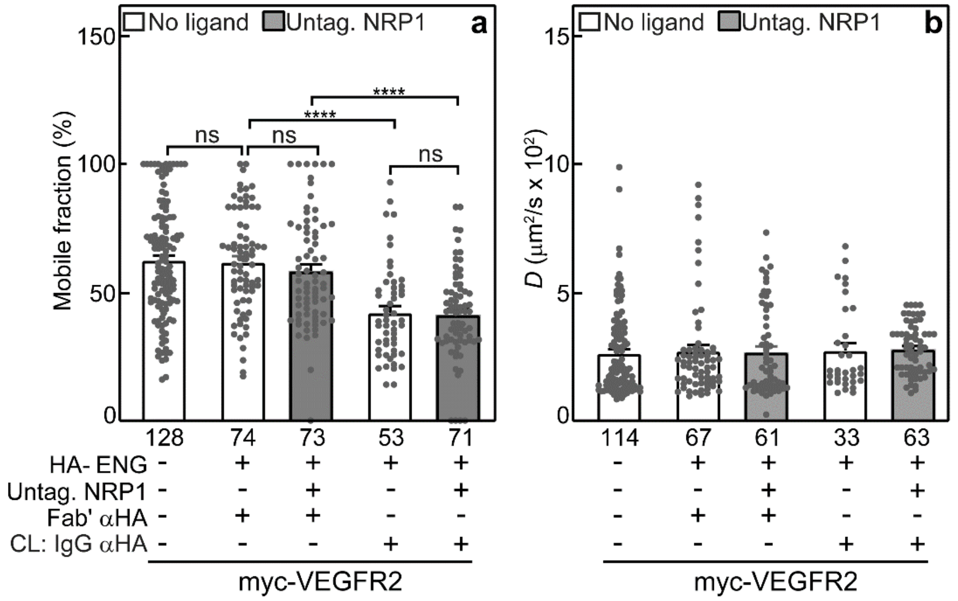

Supplementary Fig. 4 NRP1 and VEGFR2 do not compete for binding to ENG. Patch/FRAP studies were carried out on COS7 cells expressing myc-VEGFR2 alone or together with HA-ENG or with both HA-ENG and untagged NRP1 (as competitor). **a** Average R_f values; **b** Average *D* values. Bars are mean ± SEM; the number of measurements is depicted underneath each bar. R_f of myc-VEGFR2 was significantly reduced upon IgG crosslinking of HA-ENG (****, $p < 10^{-4}$; one-way ANOVA with Bonferroni post-hoc test). However, the R_f value was not significantly affected (ns = not significant) by addition of untagged NRP1 to the cotransfected vectors (compare the two rightmost bars in panel a). No significant differences (one-way ANOVA with Bonferroni post-hoc test) were found between the *D* values under any condition (b). These results are in line with the finding that ENG can bind simultaneously to NRP1 and VEGFR2 (Fig. 4), and suggests that the binding sites for the latter two receptors on ENG are not overlapping and they do not compete with each other.

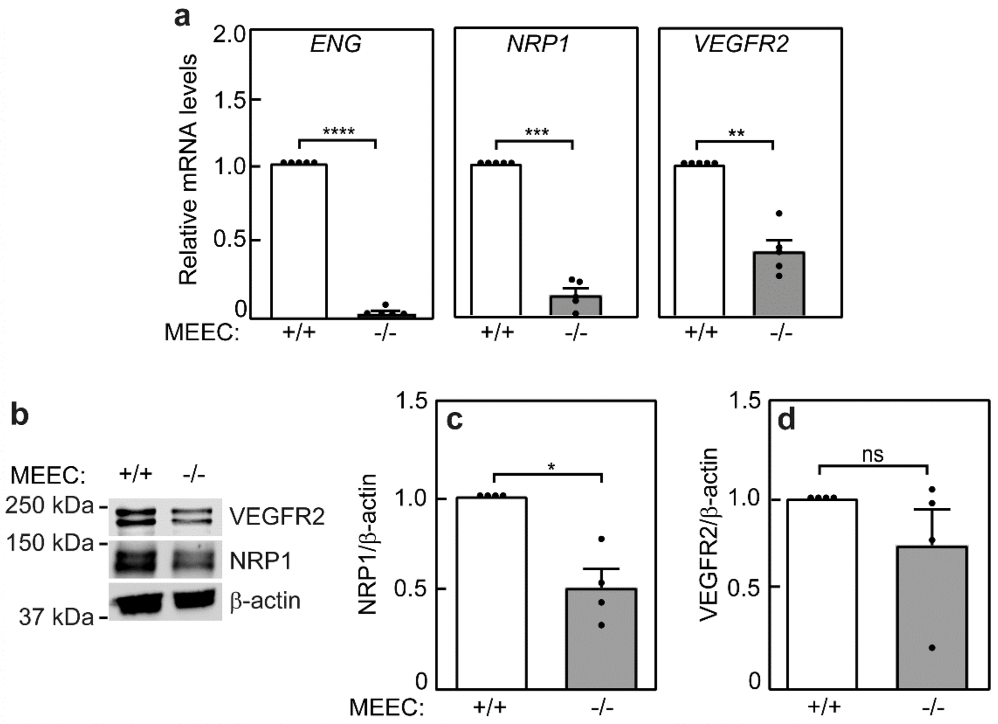

Supplementary Fig. 5 Endogenous mRNA and protein levels of ENG, NRP1 and VEGFR2 in MEEC$^{+/+}$ and MEEC$^{-/-}$ cells. a Relative mRNA levels of *ENG*, *NRP1* and *VEGFR2*. The mRNA levels were quantified using RT-qPCR, normalizing the data using mouse GAPDH as a housekeeping gene (Materials and Methods). The primers used for each receptor are listed in Supplementary Table 1. The level of the respective receptor in MEEC$^{+/+}$ was defined as 1. MEEC$^{-/-}$ showed no *ENG* expression. *VEGFR2* and *NRP1* were expressed in both cell lines, with lower levels in MEEC$^{-/-}$. **b** A typical experiment showing the relative protein levels of endogenous NRP1 and VEGFR2. Cell lysates were subjected to SDS-PAGE and immunoblotted for total NRP1 or VEGFR2, with β-actin as loading control. The bands were visualized by ECL and quantified by densitometry. **c, d** Quantification of the levels of NRP1 (c) and VEGFR2 (d). The levels of both proteins were somewhat lower in MEEC$^{-/-}$ cells, but with much less difference than that in the mRNA levels. Data are mean ± SEM from 5 independent experiments conducted in triplicates (a), or 4 independent experiments (c, d). Asterisks show significant differences between the pairs indicated by brackets, using Student's two-tailed *t*-test. *, $p < 0.05$; **, $p < 0.01$; ***, $p < 10^{-3}$; ****, $p < 10^{-4}$. ns = not significant.

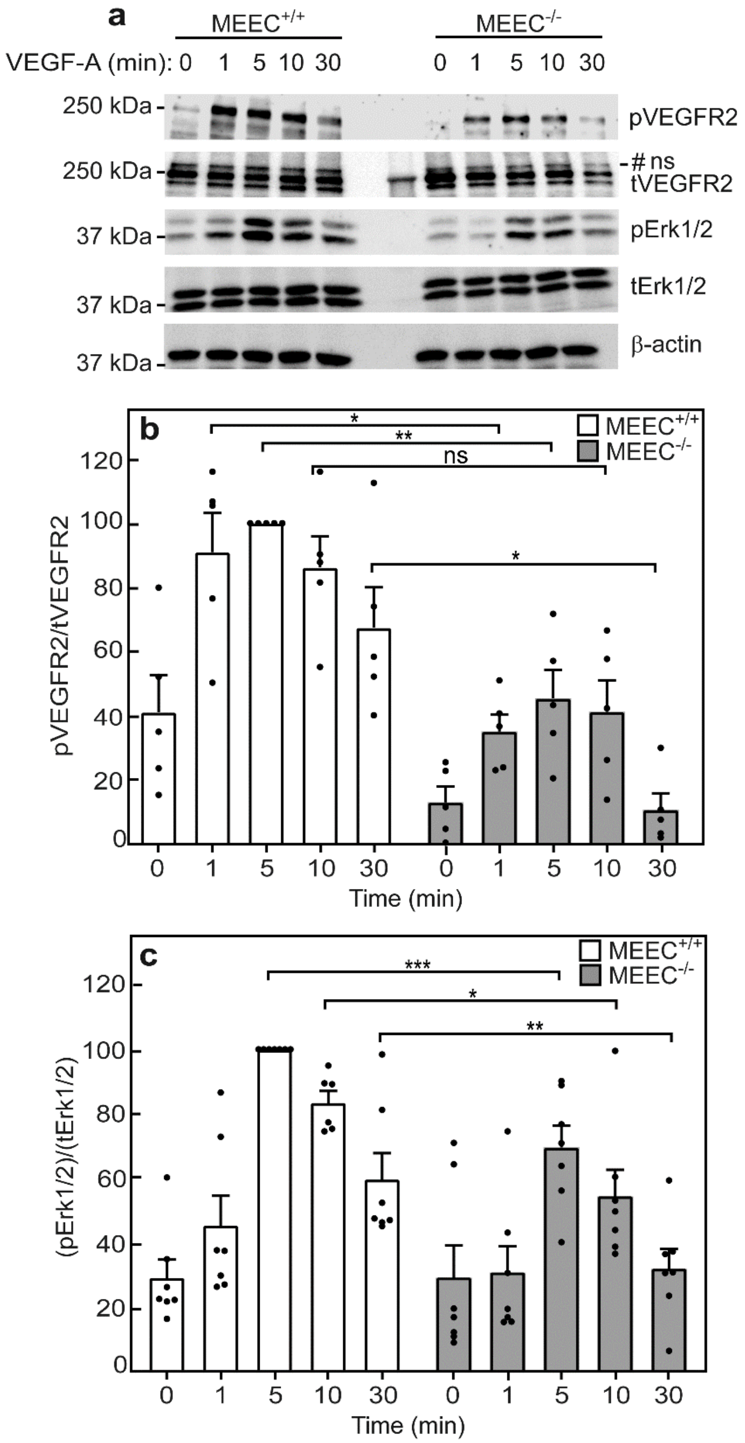

Supplementary Fig. 6 ENG enhances VEGF-A signaling to pVEGFR2 and pErk1/2 in MEECs. Cells were serum starved (30 min) and stimulated with VEGF-A (50 ng/ml) for the indicated times. Cell lysates were subjected to SDS-PAGE and immunoblotted for pVEGFR2, tVEGFR2, pErk1/2, tErk1/2 and β-actin. **a** Representative experiment. The lane to the left of the zero time in MEEC$^{-/-}$ is of the MW markers, where the 250 kDa marker is also labeled by the anti-tVEGFR2 antibody. # marks a non-specific band (ns). **b, c** Quantification of VEGF-A signaling to

pVEGFR2 (b) and to pErk1/2 (c). The bands were visualized by ECL and quantified by densitometry. Data are mean ± SEM of 5 (b) or 7 (c) independent experiments. The values obtained for MEEC$^{+/+}$ cells treated with VEGF-A for 5 min (highest values) were taken as 100%. Asterisks indicate significant differences between pairs of MEEC$^{+/+}$ and MEEC$^{-/-}$ at the same time point (one-way ANOVA and Bonferroni post-hoc test). *, $p < 0.05$; **, $p < 0.01$; ***, $p < 10^{-3}$). ns = not significant.

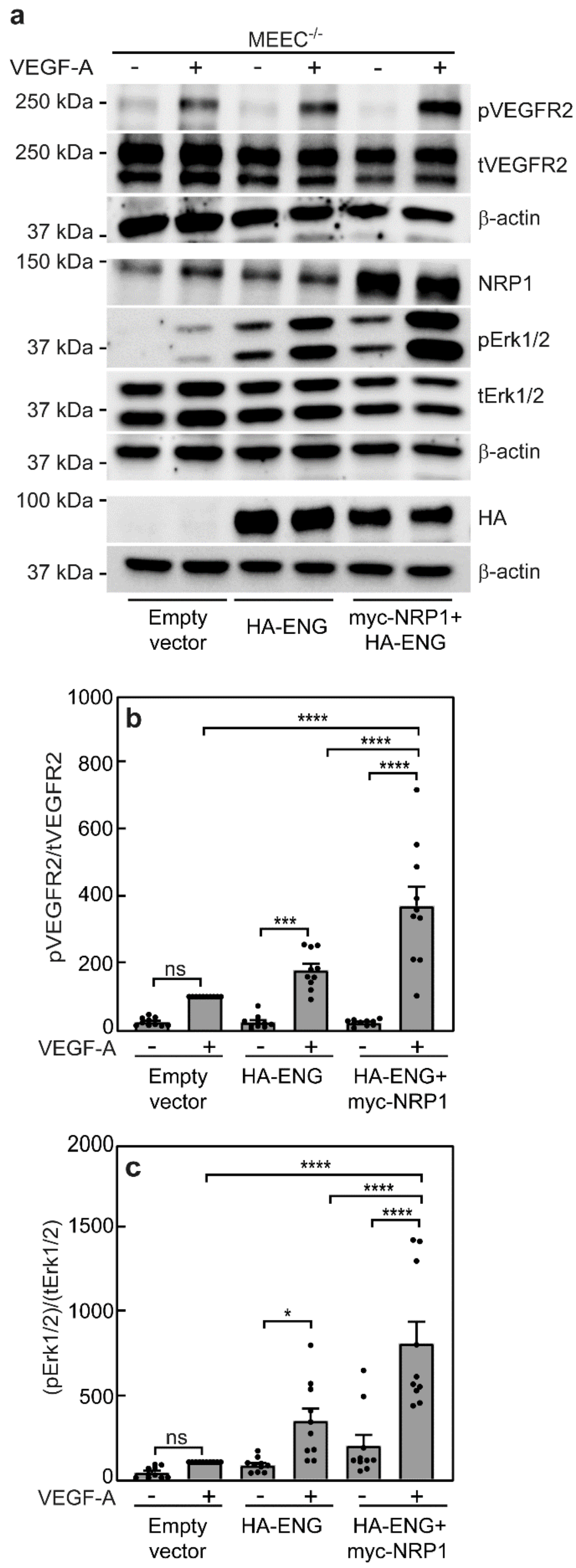

Supplementary Fig. 7 Coexpression of ENG together with NRP1 in MEEC$^{-/-}$ cells is required to enhance VEGF-A-mediated signaling. MEEC$^{-/-}$ cells were transfected with empty vector

(control), HA-ENG, or HA-ENG together with myc-NRP1 as described under Materials and Methods. After 24 h, they were serum starved (30 min) and stimulated (or not) with VEGF-A (50 ng/ml, 5 min). After lysis and SDS-PAGE, the blots were immunoblotted for pVEGFR2, tVEGFR2, NRP1, pErk1/2, tErk1/2, HA-ENG and β-actin. **a** Representative experiment. Each of the 3 groups of vertically stacked strips with its own β-actin strip as loading control was derived from a separate membrane. **b, c** Quantification of the effects of ENG (alone or together with NRP1) overexpression in MEEC$^{-/-}$ cells on VEGF-A signaling to pVEGFR2 (b) or to pErk1/2 (c). Data are mean ± SEM of 10 independent experiments. The values obtained for VEGF-A-stimulated cells transfected with empty vector were taken as 100%. Asterisks indicate significant differences between the pairs indicated by the brackets (one-way ANOVA and Bonferroni post-hoc test; *, $p < 0.05$; ***, $p < 10^{-3}$; ****, $p < 10^{-4}$). ns = not significant.

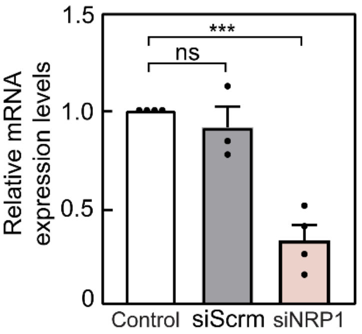

Supplementary Fig. 8 si*NRP1* transfection of MEECs effectively knocks down *NRP1* mRNA. MEEC$^{+/+}$ cells were transfected with si*NRP1* or scrambled siRNA (siScrm); untransfected cells are shown as control as well. After 48 h, the mRNA levels of *NRP1* were quantified by RT-qPCR as described under Materials and Methods. Data were normalized using mouse GAPDH as a housekeeping gene. The mRNA level of *NRP1* in untransfected MEEC$^{+/+}$ (control) was defined as 1. Data are mean ± SEM of 4 independent experiments, each performed in triplicate. Asterisks indicate significant differences between the pairs marked by brackets (one-way ANOVA and Bonferroni post-hoc test; ***, $p < 10^{-3}$). ns = not significant.

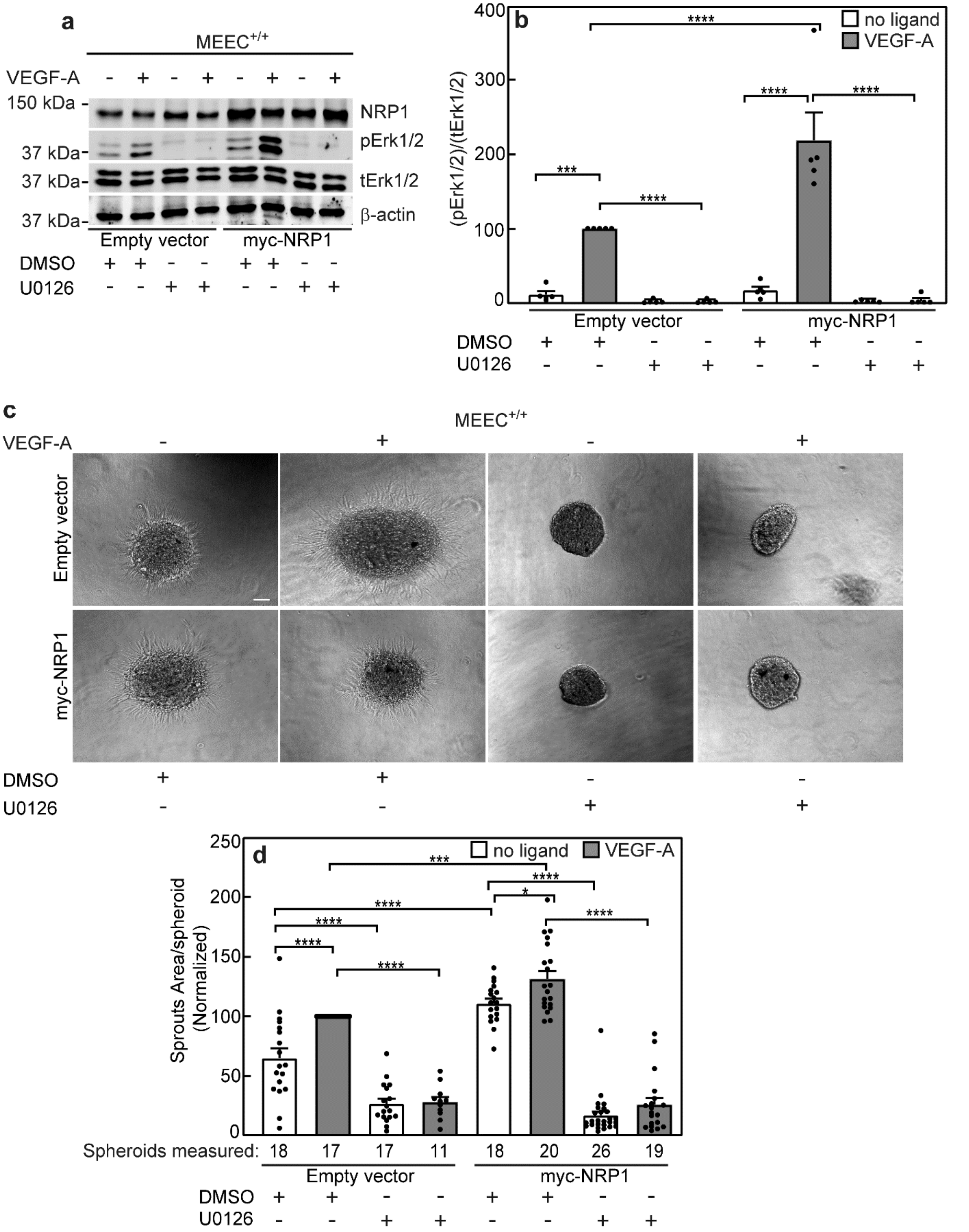

Supplementary Fig. 9 The MEK1/2 inhibitor U0126 disrupts VEGF-A signaling to pErk1/2 and sprouting in MEEC$^{+/+}$ cells with and without NRP1 overexpression. MEEC$^{+/+}$ cells were transfected by myc-NRP1 or empty vector (control). **a, b** VEGF-A signaling to pErk1/2. At 24 h

post-transfection, cells were serum starved (30 min) in medium supplemented with 1:1000 DMSO or 10 μM U0126 inhibitor, followed by stimulation with 50 ng/ml VEGF-A (5 min) where indicated. They were then immunoblotted for NRP1, pErk1/2, tErk1/2 and β-actin as in Fig. 5. **a** A representative immunoblot. **b** Quantification of the inhibition of VEGF-A-mediated pErk1/2 formation by U0126. The bands were visualized by ECL and quantified by densitometry. Data are mean ± SEM of 5 independent experiments. The values obtained for VEGF-A-stimulated MEEC$^{+/+}$ transfected with empty vector were taken as 100%. Asterisks indicate significant differences between pairs of MEEC$^{+/+}$ cells with or without myc-NRP1 overexpression (one-way ANOVA and Bonferroni post-hoc test; ***, $p < 10^{-3}$; ****, $p < 10^{-4}$). **c, d** Effects of U0126 on sprouting of MEEC$^{+/+}$ cells. At 24 h after transfection, cells were taken for sprouting experiments based on the hanging drop assay as described in Fig. 7; where indicated, U0126 (10 μM; 1:1000 from DMSO stock) or DMSO (1:1000) were added to the Matrigel on which the spheroids were allowed to sprout for another 24 h, with or without VEGF-A (100 ng/ml). **c** Typical images of spheroids subjected to the various treatments. Scale bar, 100 μm. **d** Quantification of the sprouting experiments, measured as described in Fig. 7. Sprouting was stimulated by VEGF-A, and increased further following NRP1 overexpression. U0126 treatment eliminated the formation of vascular sprouts in MEEC$^{+/+}$ cells, both without and with overexpression of NRP1. Data are mean ± SEM of n = 11-26 spheroids per condition (the number of spheroids measured are indicated under each bar) from 3 independent experiments. The area of the sprouts in VEGF-A-stimulated MEEC$^{+/+}$ was normalized to 100%, and the sprouts area under all other conditions was calculated relative to this value. *, $p < 0.05$; ***, $p < 10^{-3}$; ****, $p < 10^{-4}$ (one-way ANOVA and Bonferroni post-hoc test).

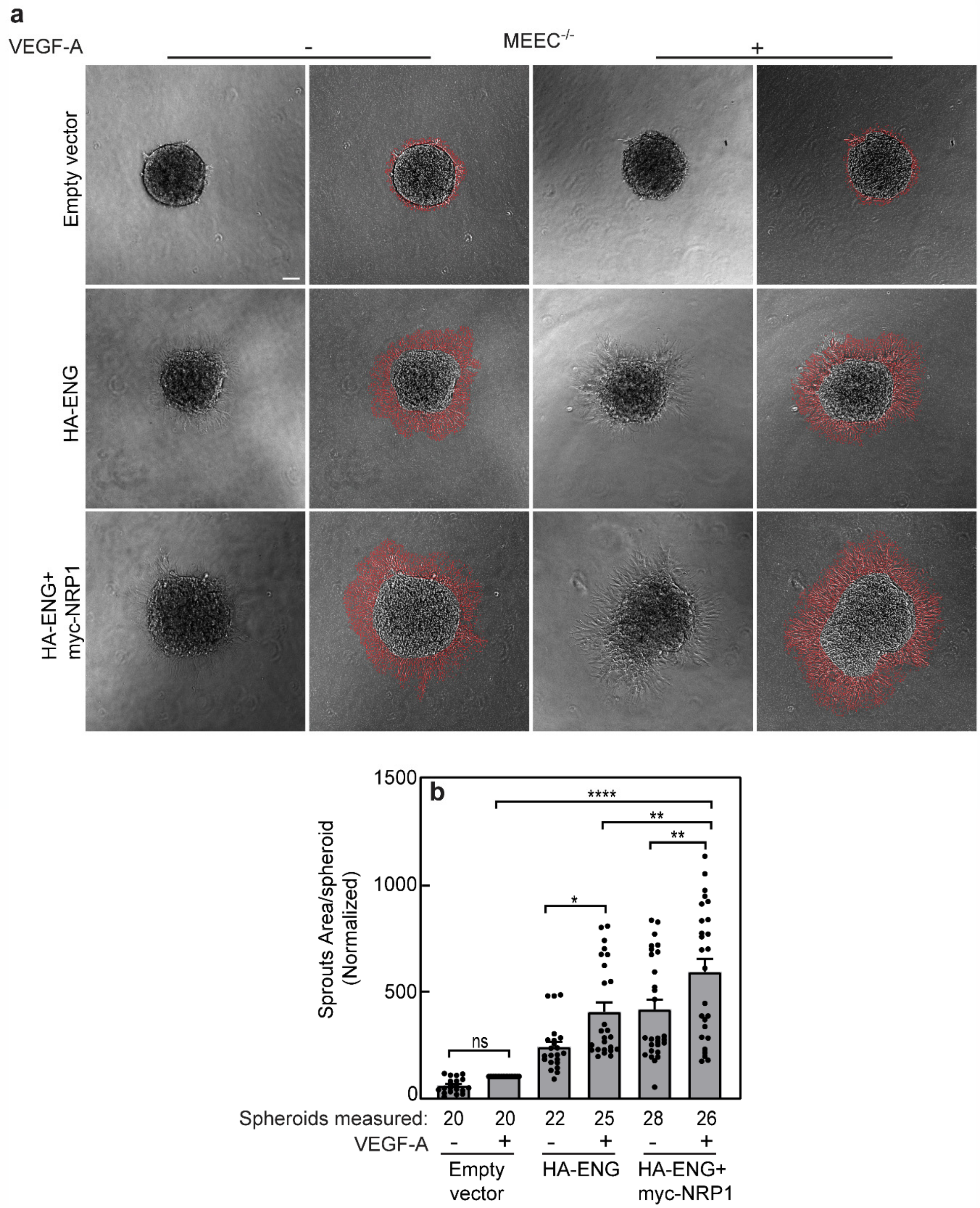

Supplementary Fig. 10 Sprouting response in MEEC$^{-/-}$ cells requires ENG and is enhanced by high levels of NRP1. MEEC$^{-/-}$ cells were transfected with empty vector (control), HA-ENG, or HA-ENG together with myc-NRP1 (Materials and Methods). After 24 h, they were taken for sprouting experiments based on the hanging drop assay as described in Fig. 7. **a** Typical images of spheroids analysed for sprouts area. The phase contrast images (left) are depicted together with the equivalent images with the outlined sprouts. Scale bar, 100 µm. **b** Quantification of the sprouting experiments. Sprouting was enabled by expression of HA-ENG in the ENG-null MEEC$^{-/-}$

cells, and was significantly enhanced upon co-transfection of myc-NRP1 together with ENG. Data are mean ± SEM of n = 19-28 spheroids per condition (n is indicated under each bar) from 3 independent experiments. The area of the sprouts in VEGF-A-stimulated $MEEC^{-/-}$ was normalized to 100%, and the sprouts area under all other conditions was calculated relative to this value. *, $p < 0.05$; **, $p < 0.01$; ****, $p < 10^{-4}$ (one-way ANOVA and Bonferroni post-hoc test). ns = not significant.

Supplementary Fig. 11 Original uncropped western blots for all signaling experiments

Prior to probing the blots by the indicated antibodies, the membranes were cut in order to allow parallel processing of the different antigens with the respective antibodies.

Fig. 5a

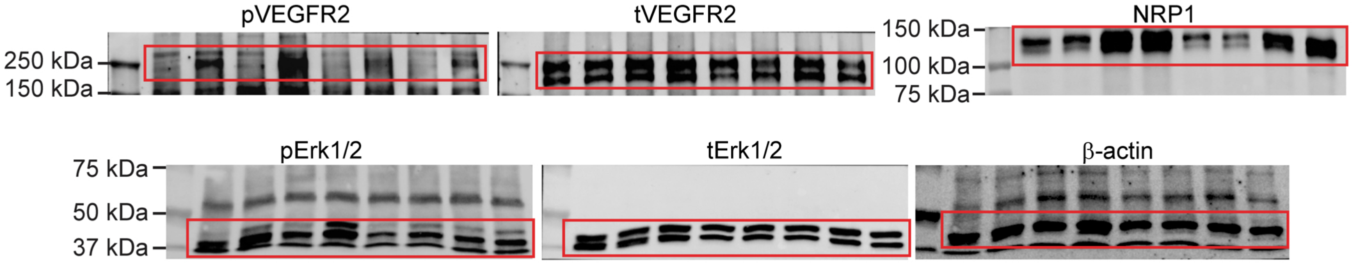

Fig. 6a

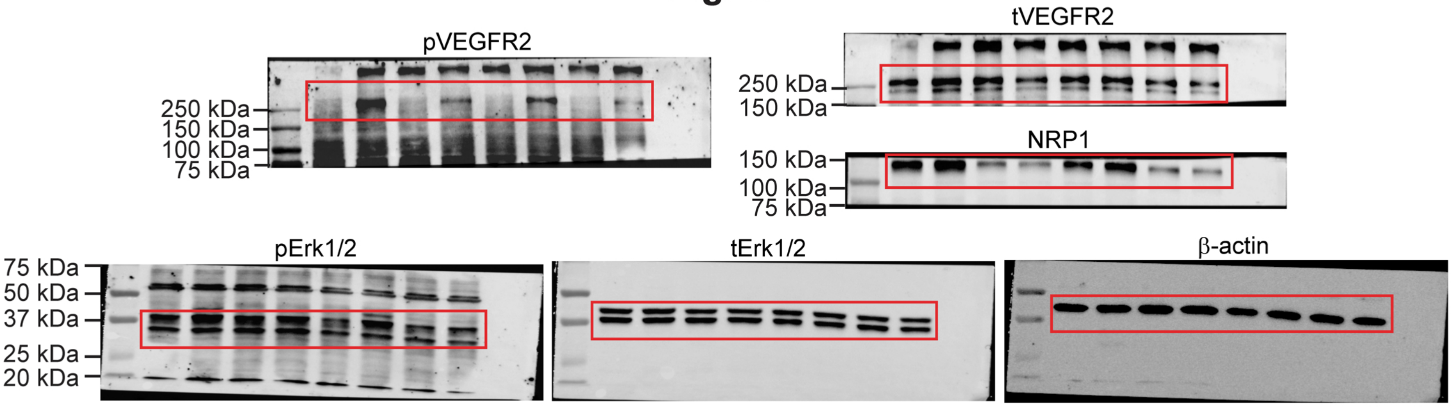

Supplementary Fig. 1a

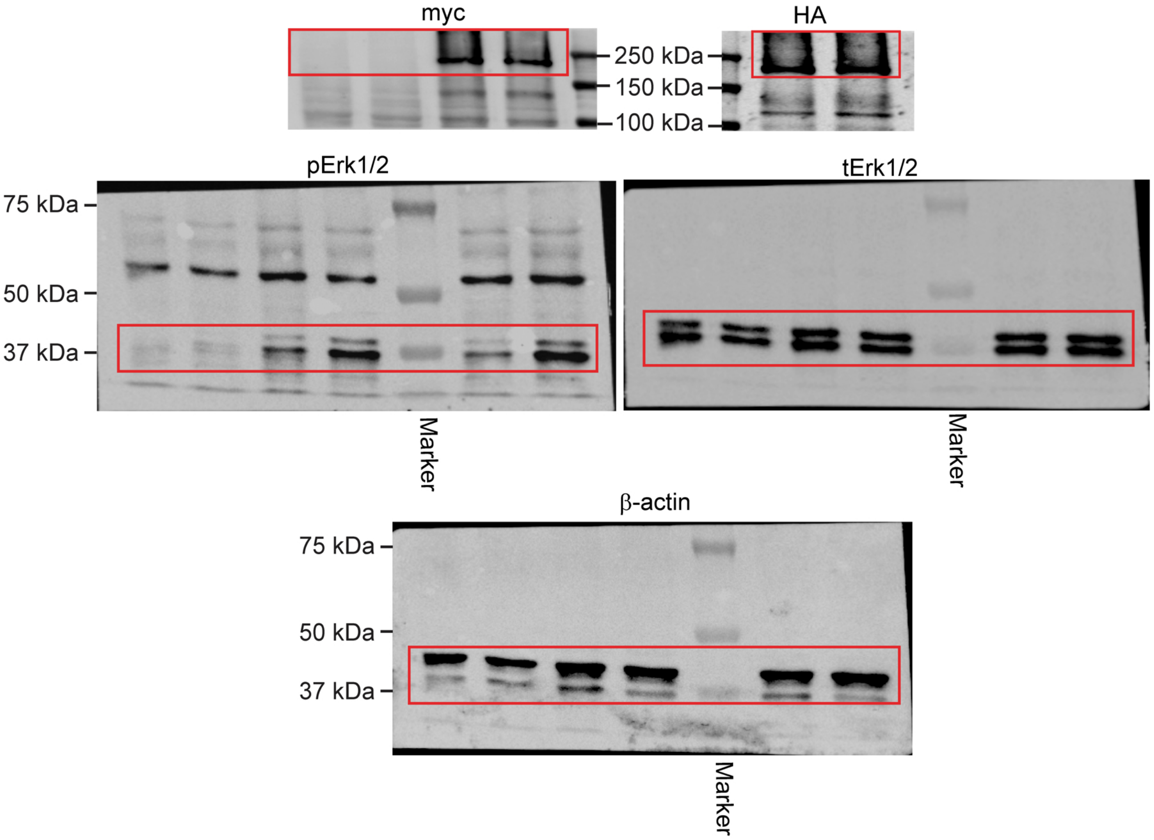

Supplementary Fig.5b

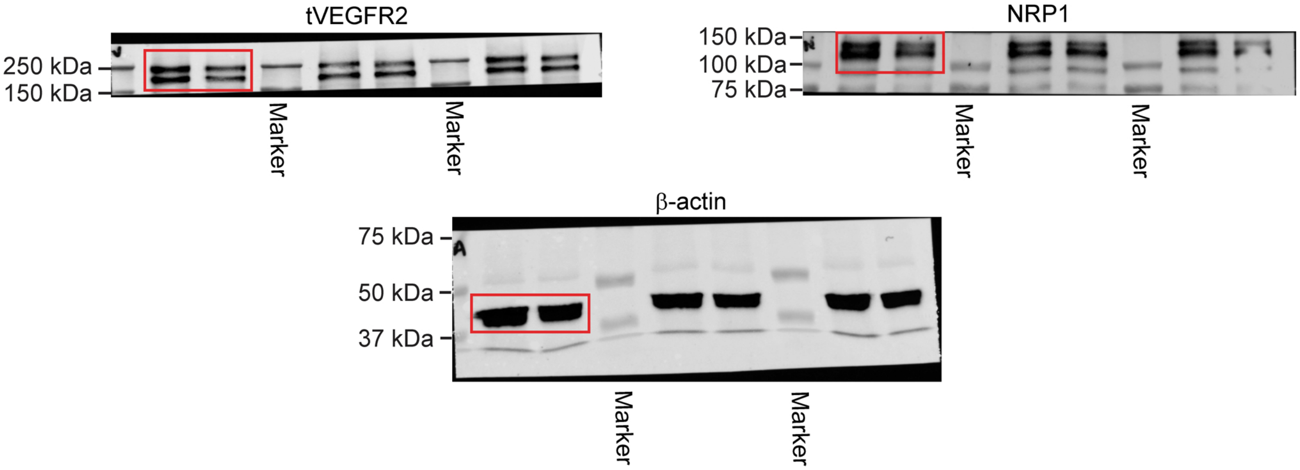

Supplementary Fig.6a

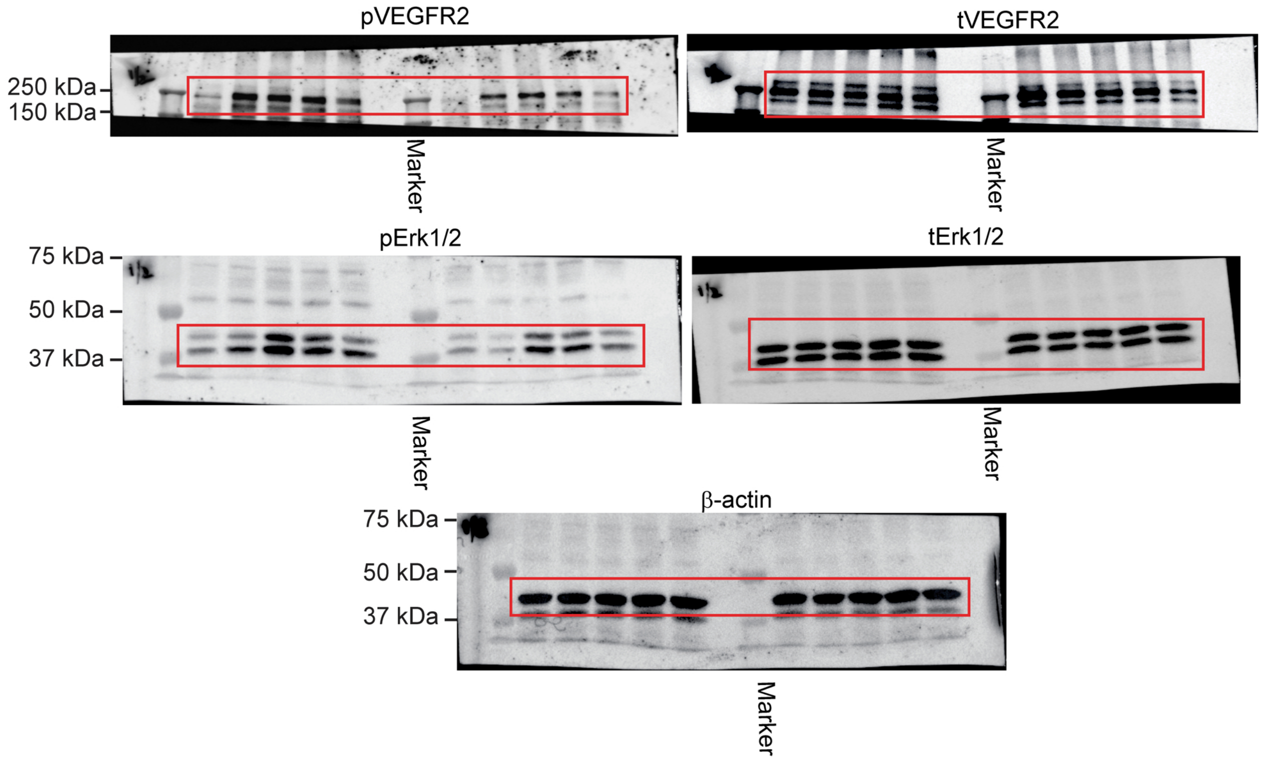

Supplementary Fig. 7a

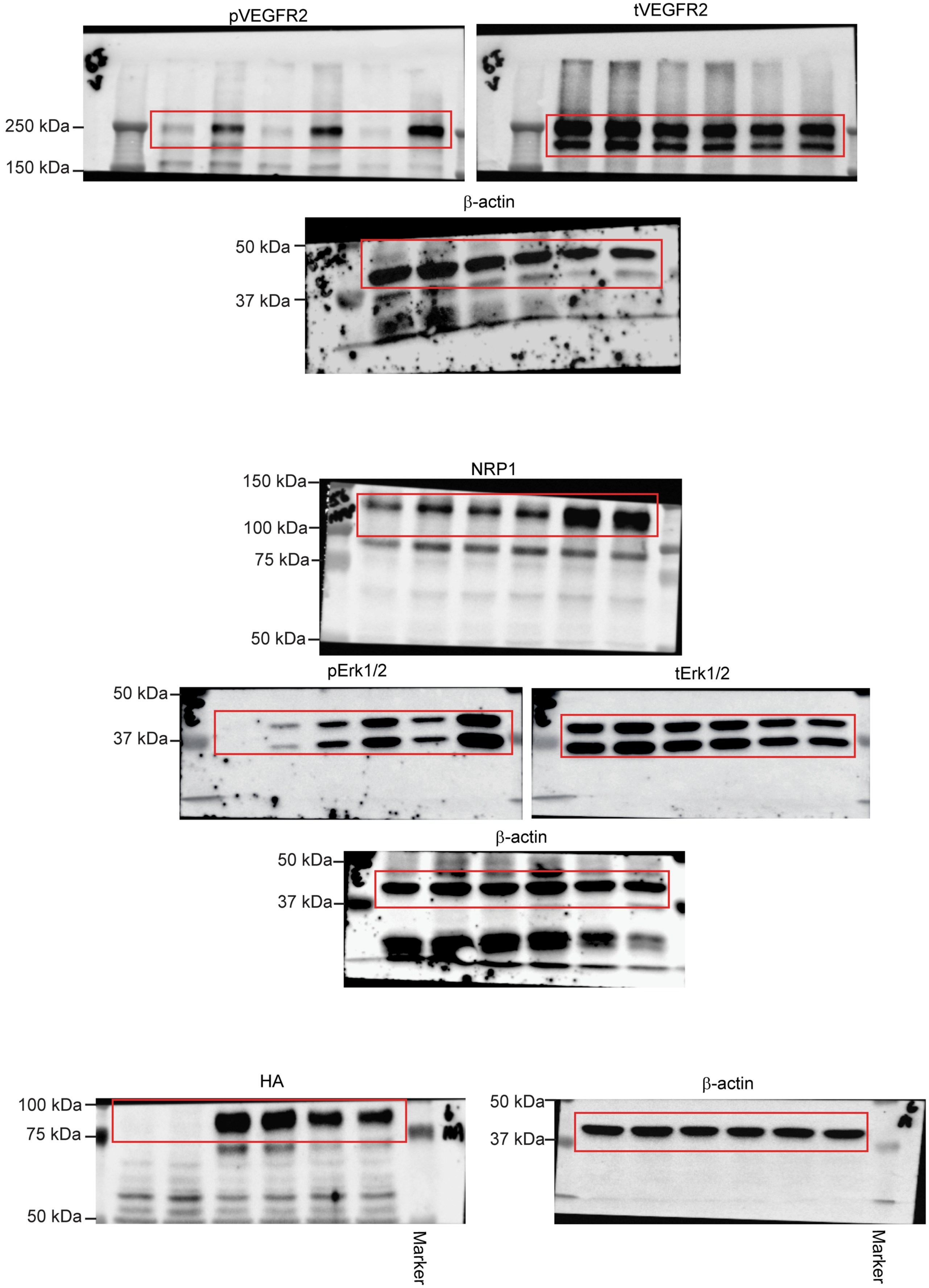

Supplementary Fig. 9a

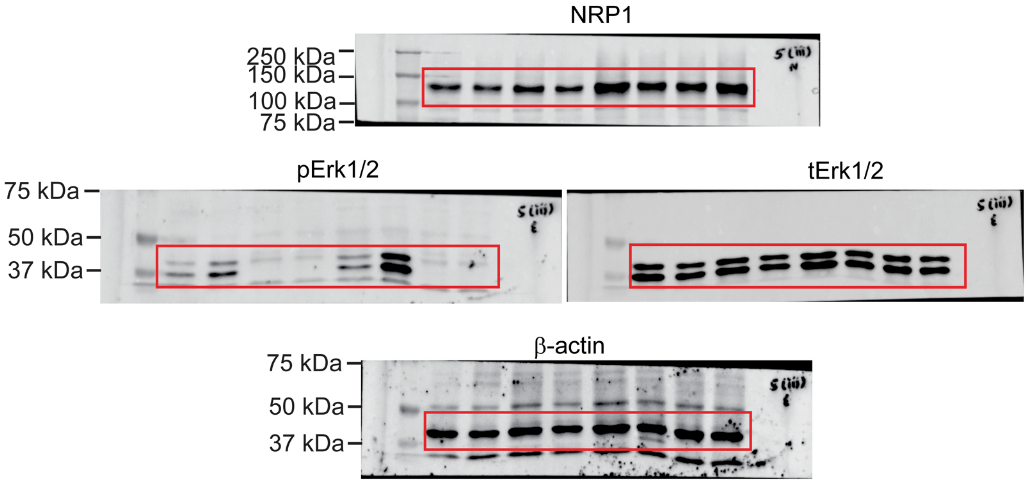

Supplement: Supplementary file 1 — Supplementary Information [file 42003_2024_5798_MOESM1_ESM.pdf]
